# Cross-modal functional connectivity supports speech understanding in cochlear implant users

**DOI:** 10.1093/cercor/bhac277

**Published:** 2022-08-20

**Authors:** Amanda M Fullerton, Deborah A Vickers, Robert Luke, Addison N Billing, David McAlpine, Heivet Hernandez-Perez, Jonathan E Peelle, Jessica J M Monaghan, Catherine M McMahon

**Affiliations:** Department of Linguistics and Macquarie University Hearing, Australian Hearing Hub, Macquarie University, Sydney 2109, Australia; Cambridge Hearing Group, Sound Lab, Department of Clinical Neurosciences, University of Cambridge, Cambridge CB2 OSZ, United Kingdom; Speech, Hearing and Phonetic Sciences, University College London, London WC1N 1PF, United Kingdom; Department of Linguistics and Macquarie University Hearing, Australian Hearing Hub, Macquarie University, Sydney 2109, Australia; Institute of Cognitive Neuroscience, University College London, London WCIN 3AZ, United Kingdom; DOT-HUB, Department of Medical Physics and Biomedical Engineering, University College London, London WC1E 6BT, United Kingdom; Department of Linguistics and Macquarie University Hearing, Australian Hearing Hub, Macquarie University, Sydney 2109, Australia; Department of Linguistics and Macquarie University Hearing, Australian Hearing Hub, Macquarie University, Sydney 2109, Australia; Department of Otolaryngology, Washington University in St. Louis, St. Louis, MO 63110, United States; National Acoustic Laboratories, Australian Hearing Hub, Sydney 2109, Australia; Department of Linguistics and Macquarie University Hearing, Australian Hearing Hub, Macquarie University, Sydney 2109, Australia; Department of Linguistics and Macquarie University Hearing, Australian Hearing Hub, Macquarie University, Sydney 2109, Australia; HEAR Centre, Macquarie University, Sydney 2109, Australia

**Keywords:** cochlear implant, cross-modal plasticity, functional near-infrared spectroscopy, functional connectivity, speech perception

## Abstract

Sensory deprivation can lead to cross-modal cortical changes, whereby sensory brain regions deprived of input may be recruited to perform atypical function. Enhanced cross-modal responses to visual stimuli observed in auditory cortex of postlingually deaf cochlear implant (CI) users are hypothesized to reflect increased activation of cortical language regions, but it is unclear if this cross-modal activity is “adaptive” or “mal-adaptive” for speech understanding. To determine if increased activation of language regions is correlated with better speech understanding in CI users, we assessed task-related activation and functional connectivity of auditory and visual cortices to auditory and visual speech and non-speech stimuli in CI users (*n* = 14) and normal-hearing listeners (*n* = 17) and used functional near-infrared spectroscopy to measure hemodynamic responses. We used visually presented speech and non-speech to investigate neural processes related to linguistic content and observed that CI users show beneficial cross-modal effects. Specifically, an increase in connectivity between the left auditory and visual cortices—presumed primary sites of cortical language processing—was positively correlated with CI users’ abilities to understand speech in background noise. Cross-modal activity in auditory cortex of postlingually deaf CI users may reflect adaptive activity of a distributed, multimodal speech network, recruited to enhance speech understanding.

## Introduction

Cochlear implants (CIs) are a transformative technology, providing users with dysfunctional inner ears the ability to communicate using spoken language. CIs bypass the damaged or absent sensory cells of the inner ear to stimulate directly the auditory nerve fibers with trains of electrical pulses, processed to convey the energy envelopes of speech sounds (Wilson and Dorman 2008; Macherey and Carlyon 2014). Nevertheless, despite their acknowledged success as an effective therapeutic intervention for severe-to-profound hearing loss (Carlyon and Goehring 2021), speech understanding following cochlear implantation can be highly variable (Blamey et al. 2013) and remains significantly poorer relative to individuals with normal hearing (NH) (Firszt et al. 2004), even after clinical optimization of device parameters (Green et al. 2007; Heydebrand et al. 2007; Gifford et al. 2008). In the case of postlingually deaf CI recipients, i.e. those who lose their hearing after developing speech and language abilities, restoring the sensation of sound does not always translate to high levels of speech recognition (Blamey et al. 2013; Holden et al. 2013; Boisvert et al. 2020). Given the relatively coarse spectral content of CI signals compared to acoustic hearing (Wilson and Dorman 2008), postlingually deaf CI recipients need to form new associations between sounds and their meaning after implantation, which often demands additional neural resources, such as cognitive load, particularly in challenging listening environments (Peelle 2018; Sherafati et al. 2022), as well as ongoing use of additional cues to speech understanding such as lipreading (Giraud et al. 2001; Stropahl et al. 2015; Stropahl and Debener 2017).

In seeking to explain the variation in performance outcomes between CI users, less than a quarter of which is explicable by patient-specific factors or issues associated with the integrity of the peripheral auditory structures (Blamey et al. 2013), interest has focused on understanding the consequences of hearing loss on higher brain functions. This includes the possibility that neuroplasticity—adaptive and *mal*-adaptive changes to the structure and/or function of cortical centers that follow periods of sensory deprivation—contributes to speech understanding following cochlear implantation (Stropahl et al. 2017; Anderson, Lazard, et al. 2017a). Cross-modal plasticity, whereby alternate sensory modalities “take over” a deprived sense’s cortical representation leading to enhanced perception of that sense, e.g. heightened auditory acuity in blindness (Bavelier and Neville 2002), has been proposed as one factor that might limit speech understanding following implantation. However, while such compensatory responses are well-evidenced in cases of congenital deafness in animals (Lomber et al. 2010) and in humans (Kral 2007), it remains unclear whether such a fundamental reorganization of brain function occurs in postlingually acquired deafness in humans, and if so, how this might influence hearing and listening abilities following cochlear implantation (Luke et al. 2015; Anderson, Lazard, et al. 2017a; Stropahl et al. 2017; Gransier et al. 2020).

One factor that might influence the interpretation of studies designed to assess cross-modal plasticity in CI listeners is the range of auditory and visual stimuli employed—including the use of speech versus non-speech material. Increased auditory cortical activity to non-speech visual stimuli such as checkerboards or gratings has been associated with poorer speech understanding in CI listeners (Doucet et al. 2006; Sandmann et al. 2012; Chen et al. 2016), suggesting that “visual takeover” of auditory cortex following hearing loss may limit the capacity for functional recovery, post-implantation (Stropahl et al. 2017). Conversely, a positive association has been reported between increased cross-modal activity to visually presented sentences and auditory speech understanding, prior to, and 6 months after, cochlear implantation in postlingually deaf adults (Anderson, Wiggins, et al. 2017b). In addition, cross-modal activation of auditory cortex in CI users has been associated with increased audiovisual (AV) integration, which is proposed to support multisensory aspects of speech communication (Stropahl and Debener 2017). Differences in cross-modal influences—from detrimental to beneficial—therefore, might be explained by a more nuanced model of speech processing, including its manifestation at the cortical level in terms of a holistic representation of communication and language, rather than solely a series of acoustic events.

Neuroimaging studies indicating a gradation of cortical activations to hierarchical auditory stimuli demonstrate more widespread brain activations with increasing acoustic and linguistic demand (see Peelle 2012 for review). This suggests that the choice of visual stimuli employed when investigating cross-modal plasticity may be important. Visual speech—relevant for speech processing in everyday listening tasks where visual information can be leveraged for speech understanding—has been shown to engage left temporal regions even in normal-hearing (NH) individuals (Calvert et al. 1997; MacSweeney et al. 2002; Hall et al. 2005). To date, however, no study has directly examined stimulus-specific effects (i.e. non-speech vs. speech stimuli) in the same group of CI users or compared this to an age-matched, NH population.

Here, using functional near-infrared spectroscopy (fNIRS) to measure changes in regional blood oxygenation related to neural activity, we assessed stimulus-related differences in cross-modal activation and task-related functional connectivity between auditory and visual cortical regions, in a cohort of postlingually deaf CI users, and compared these differences to those of age-matched, NH listeners. Consistent with current models of language processing (Hickok and Poeppel 2007, 2015), we tested the hypothesis that speech and non-speech stimuli engage different functional networks and that, specifically, visual speech recruits distinct regions within a distributed, multimodal network for speech processing, particularly within superior temporal cortical regions. Our data demonstrate that auditory cortical areas are activated by speech, regardless of the mode of presentation (auditory or visual), and that cross-modal, visual-evoked activity in auditory cortex depends on stimulus type (i.e. speech vs. non-speech) in CI users. Importantly, our data also show that stimulus-based differences in activation patterns and task-related interregional connectivity are correlated with behavioral measures of speech understanding in CI users, but not NH listeners. Together, they support the notion of a multimodal speech processing network that represents coordinated activity between different brain regions, and that high-performing CI users leverage this network to enhance their capacity for listening in noise. Cross-modal activation of auditory cortex in postlingually deaf adults who use CIs for hearing may reflect their enhanced capacity to leverage an intact cortical language network to improve listening abilities, rather than any form of maladaptation in response to sensory deprivation.

## Materials and methods

The study was approved by the University College London Divisional Ethics Committee of Psychology and Language Sciences (reference ID: SHaPS-2018-DV-028), and written informed consent was obtained from all participants.

### Participants

All participants were native adult English speakers and had self-reported normal or corrected vision and no cognitive impairment. Two groups of participants were recruited. The first group consisted of 20 adults (mean age = 70 years, SD = 6.88, range 53–79 years) with hearing within normal limits, defined as 4-frequency pure-tone average (0.5, 1, 2, 4 kHz) air conduction thresholds of ≤25 decibels (dB HL) bilaterally. Three participants were excluded from the final data analysis due to poor data quality (low signal to noise ratio), which may have arisen from poor contact between fNIRS optodes and the scalp, or excessive movement during testing. The second group consisted of 15 adults (mean age = 68 years, SD = 6.58, range 54–80 years) with postlingually acquired bilateral severe-to-profound deafness, and ≥12 months of CI experience (mean years of bilateral deafness = 19.8, SD = 15.98, range 4–68 years). One CI subject was not able to participate in both test sessions; therefore, this subject was excluded from the final analyses. Three participants were bilaterally implanted, and 7 of the remaining 12 were implanted in the right ear. Where the subject was bilaterally implanted, we followed the user’s preferential side, and this was predominantly right. All CI subjects had a pure tone average (PTA, 0.5, 1, and 2 kHz) greater than 90 dBHL in the contralateral ear (mean PTA = 108 dB, SD = 9.64). All participants were right-handed as measured by the Edinburgh Handedness Inventory (Oldfield 1971), except for one NH participant. Participant characteristics are summarized in Table 1.

**Table 1:** Participant characteristics.

ID	Grp	Age (years)	Sex (M/F)	Handedness (L or R)	Implant ear/s (L or R) - CI duration (years)	HA use in contralateral ear? (Y or N)	Hearing in contralateral ear (PTA, dBHL)	S/P deafness (years)	HA use (years)	A-only (%)	A-only speech-in-noise score (SRT)	V-only score (%)
1	CI	67	F	R	R-10	Y	117	12	~25	99	−1.8	12
2	CI	74	F	R	R-13, L-15	N	120	22	~31	94	−0.33	48
3	CI	71	F	R	L-15	Y	110	68	57	92	3.14	14
4	CI	76	F	R	R-6, L-6	N	120	36	~36	100	−0.14	10
5	CI	72	M	R	R-4	N	117	9	15	76	1.5	3
6	CI	54	M	R	L-4	Y	113	9	14	5	6.43	12
7	CI	71	F	R	R-5	N	110	11	10	93	1.33	12
8	CI	73	F	R	R-3	N	102	18	34	94	1.52	10
9^a^	CI	68	F	R	R-13	Y	102	17	61	95	1.6	49
10	CI	80	F	R	L-3	Y	98	4	68	25	11	36
11	CI	67	F	R	L-7	N	97	12	28	100	−5.33	11
12	CI	65	F	R	R-22	N	102	23	40	97	0.89	36
13	CI	61	M	R	R-19, L-25	Y	120	27	NA	8	7.5	35
14	CI	67	F	R	L-3	Y	97	5	38	96	3	18
15	CI	61	F	R	R-1.5	N	93	23	20	90	−1	4
1	NH	66	F	R	−	−	−	−	−	100	−9.6	2
2	NH	72	M	R	−	−	−	−	−	100	−12.24	7
3	NH	79	M	R	−	−	−	−	−	100	−12	0
4	NH	76	F	R	−	−	−	−	−	99	−6.04	13
5	NH	63	F	L	−	−	−	−	−	100	−14.22	5
6	NH	66	F	R	−	−	−	−	−	100	−15.22	3
7	NH	79	F	R	−	−	−	−	−	100	−9.25	4
8	NH	67	M	R	−	−	−	−	−	100	−11.36	3
9	NH	66	F	R	−	−	−	−	−	100	−11.77	3
10	NH	66	F	R	−	−	−	−	−	99	−10.5	32
11	NH	68	F	R	−	−	−	−	−	100	−11.04	4
12	NH	69	M	R	−	−	−	−	−	100	−9.3	6
13	NH	79	F	R	−	−	−	−	−	100	−12.29	18
14	NH	72	F	R	−	−	−	−	−	100	−11	9
15	NH	76	M	R	−	−	−	−	−	99	−8	12
16	NH	79	M	R	−	−	−	−	−	100	−13.48	1
17	NH	53	M	R	−	−	−	−	−	100	−15.75	12

Table column headings refer to: subject identification (ID); study group (Grp); age in years at time of testing (Age (years)); sex (Sex (M/F)); dominant hand (Handedness (L or R)); side of implantation (L—left, R—right) and years of implant use (Implant ear/s (L or R), CI duration); Bimodal, unilateral, or bilateral CI user (Hearing aid used in contralateral ear? (Y or N)); PTA (0.5–2 kHz) hearing level in contralateral ear (Hearing in contralateral ear (PTA, dBHL)); number of years of bilateral severe to profound deafness (S/P deafness (years)); number of years of hearing aid use (HA use (years)); auditory-only in quiet speech understanding score, percentage correct (A-only (%)); auditory-only speech-in-noise understanding, auditory speech reception threshold (SRT) (A-only speech-in-noise score (SRT)); visual-only (lipreading) score, percentage correct (V-only score (%)).

^a^Subject excluded from analysis.

### Behavioral test paradigm

Sentences from the Institute of Electrical and Electronics Engineers (IEEE) corpus (Rothauser et al. 1969) recorded in British English were used to assess speech understanding in auditory-only, visual-only, and AV conditions. The corpus comprises 72 lists of 10 sentences each, described as the “1965 Revised List of Phonetically Balanced Sentences (Harvard Sentences).” The sentences are phonetically balanced and use specific phonemes at the same frequency that they appear in English. Each sentence consisted of 5 key words, for example “Always close the barn door tight.” Two lists (a total of 20 sentences) were presented per condition and presentation was randomized across participants. Participants were instructed to repeat as many words as they were able to identify, and items were scored for whole key words correct. Given the likelihood of ceiling effects in comparing the 2 groups with auditory speech tasks in quiet, the Children’s Coordinate Response Measure (CCRM) was used as a speech-in-noise task, based on a test developed by Bolia et al. (2000) and Brungart (2001), which was selected for its low contextual cues. Sentences had a set structure, e.g. “Show the dog where the (color) (number) is?”: e.g. “Show the dog where the green three is?”, with 6 color options (blue, black, green, pink, red, and white) and 8 possible numbers (1–9, excluding 7). The stimuli were sentences spoken by a native British female speaker, presented at 70 dB SPL, in 2-talker babble noise, the signal-to-noise ratio (SNR) of which was adjusted adaptively depending on the accuracy of responses, i.e. an incorrect response led to one step up, and a correct response, one step down, in order to track the 50% correct threshold. The starting SNR was 20 dB, with an initial step size of 5 dB SNR, that decreased after 2 reversals to 2 dB. The final SNR was calculated as the mean of the last 4 reversals, as per the procedure outlined in de Kerangal et al. (2021). The maximum number of trials did not exceed 25. The metric of AV speech advantage or “visual enhancement” was calculated for individual subjects as “AV score – A only score,” to determine additional benefit from the added visual speech signal to the auditory signal (Sommers et al. 2005). In view of the non-normal distribution of the data, differences in performance between groups (i.e. percentage of correct responses) were analyzed using a nonparametric Wilcoxon rank-sum test.

### Functional near-infrared spectroscopy imaging

fNIRS was employed as a noninvasive, optical imaging technique that has been shown to be suitable for the study of cortical activity in CI participants (Saliba et al. 2016) and, more broadly, language and cognitive neuroscience research (Quaresima et al. 2012; Pinti et al. 2020). Given that it uses light, fNIRS is therefore not subject to the same electric or magnetic artifacts that limit other available neuroimaging techniques, when considering experiments with users of hearing devices. It is also quiet, particularly in comparison with fMRI, thus making it ideal for auditory experiments. fNIRS uses near-infrared light directed onto the scalp, in order to measure cortical hemodynamic based on changes in transmission of light through the scalp and skull and surface of the brain—and provides a proxy measure for neural activation.

### fNIRS experimental paradigm and conditions

**Fig. 1:**
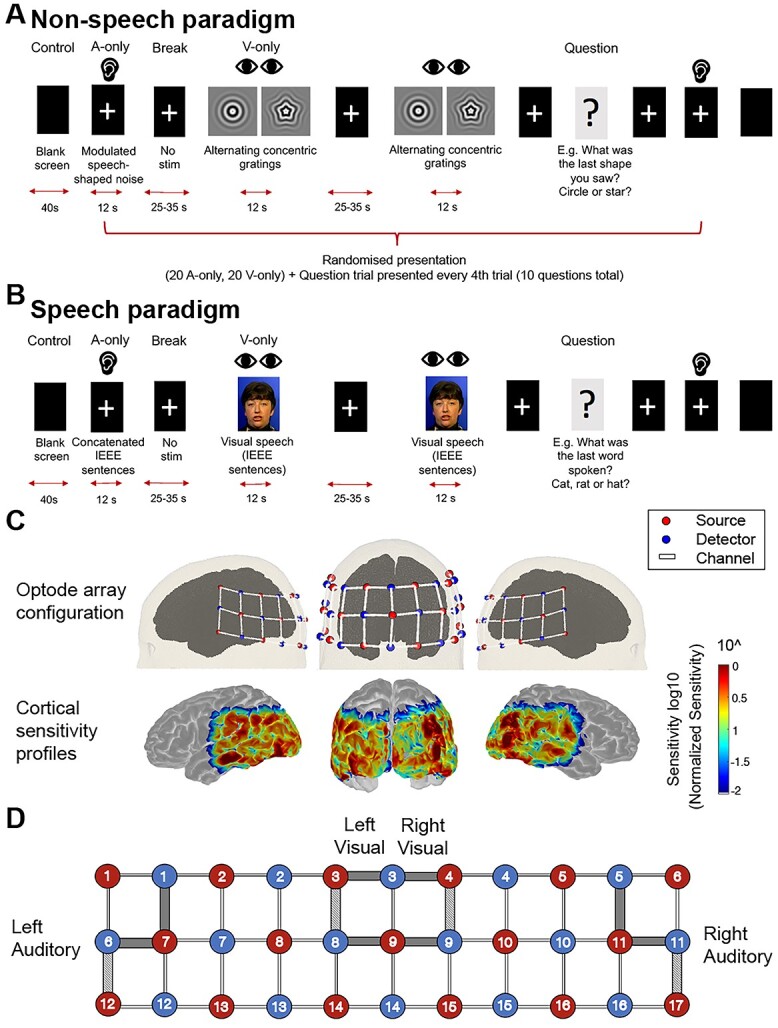
fNIRS experimental block design and cortical coverage of ROIs. A) Schematic of the “non-speech” paradigm, with “low-level” auditory (modulated speech-shaped noise) and visual stimuli (alternating concentric gratings) and B) Schematic of the “speech” paradigm, with concatenated sentence stimuli (IEEE corpus). All rectangles represent what the subject was viewing on the screen. A fixation cross was always present in the center of the screen (apart from control, “blank screen” trials). C) fNIRS 52 channel (17 sources, 16 detectors) optode array configuration positioned over auditory and visual ROIs (top) and sensitivity profile of probe to underlying cortical regions, generated in AtlasViewer (bottom). ROIs include bilateral superior temporal cortices and occipital cortices. D) Two-dimensional representation of fNIRS optode array, with gray shaded channels indicating channel selections forming the 4 ROIs for correlation analyses with behavioral measures. Within these shaded channels, the light gray shading additionally indicates seed channels used in the correlational analyses between coherence values and behavioral measures of speech understanding.

Two manipulations (“non-speech” and “speech”) of a block design paradigm were administered over 2 sessions (Fig. 1A and B), with a total of 80 block trials across the 2 manipulations of the block design paradigm—20 trials each of auditory or visual, speech or non-speech stimuli. The blocks were interleaved with breaks of 25–35 s randomized duration, as well as “attention” trials every fourth trial, which included a 2 or 3 alternate forced choice question. The questions asked about what was seen or heard in the preceding trial, e.g. “What was the last shape you saw?” or “Did you hear noise in the previous trial?” for the non-speech paradigm, or “What was the last word spoken?” for the speech paradigm (Fig. 1B). For both paradigms, the order of presentation was pseudo-randomized, whereby question trials occurred every fourth trial, and the order of remaining auditory and visual trials was randomized across participants. During rest periods, a white fixation cross was presented on a black screen, which remained present throughout the auditory-only condition. This fixation cross was used to help the subject maintain a consistent gaze and therefore head positioning, as well as encouraging attention to the task (Thielen et al. 2019). Fixation crosses have been employed in previous studies during auditory stimulation blocks (Anderson, Lazard, et al. 2017a; Shader et al. 2021). For question trials, a written question appeared on the black screen with alternative choice answers and participants were instructed to indicate their answer via button press on a keyboard. An additional 10 s was added before and after the subsequent rest period. This paradigm was piloted and refined to ensure that it was of reasonable duration for participants, in order to reduce potential task fatigue and impacts to sustained attention, which has been shown to occur for tasks longer than an hour in duration (Sarter et al. 2001). Further, the tester was always present in the room for the entire duration of the experiment to monitor aspects such as physical movement and attention to the task.

Stimuli for both fNIRS paradigms were presented using Presentation software (Neurobehavioral Systems, Inc., Berkeley, CA). Participants were seated in front of a computer monitor at a comfortable viewing distance of approximately 1 m. For NH participants, all auditory stimuli were presented via ER-1 insert earphones to the right ear only, with contralateral ear plugged. For the CI participants, an Otocube (Otoconsult NV, Antwerp, Belgium)—a custom-built, magnetically shielded loudspeaker designed for use with CI participants, was used to deliver auditory stimuli, where an extended coil cable was attached to the speech processor, which allowed the speech processor to be enclosed in the shielded chamber with mini loudspeaker. This method of delivery ensured stimulation of the CI ear only, without interference or assistance from the contralateral ear. In the case of the bilateral CI users, 2 were presented with auditory stimuli in the right CI, and 1 in the left CI. The sound pressure level (SPL) of the auditory stimuli delivered to both groups was calibrated using a Brüel and Kjaer Photon dynamic signal analyzer and artificial ear, calibrated to a comfortable listening level of 70 dB SPL.

Instructions for each paradigm were delivered both verbally and in written form prior to measurement. For both paradigms, participants were instructed to always fixate on the center of the screen and to remain still for the duration of the test session, which was around 35 min in total. For the speech paradigm, participants were instructed to attend to the talker and try to understand what was said, in both the visual and auditory modalities (presented separately).

### Non-speech stimuli

Blocks of speech-shaped noise with the same spectral content as the speech corpus, with a modulation frequency of 0.7 Hz (to match the visual non-speech stimulus), were presented as the auditory condition. These auditory blocks were 12 s in duration. For the visual condition, the stimuli consisted of a high-contrast, sinusoidal concentric grating which alternated with a similarly radially modulated star-shaped grating (Fig. 1A) for a duration of 700 ms each, adapted from Doucet et al. (2006). The circle and star stimuli were presented for an equivalent duration of the auditory stimuli, 12 s, with the intention of inducing the percept of shape transformation, which has been shown to activate the ventral visual pathway in humans (Doucet et al. 2005, 2006). Participants were instructed to focus their gaze on the center of the concentric circle/star pattern for the duration of the testing. This paradigm was particularly selected for use as a “low-level” stimulus that bears no relationship to linguistic elements in terms of temporal features, with the intention of being in contrast with the more ecological, speech-based manipulation of the paradigm described below.

### Speech stimuli

Blocks of 4 concatenated IEEE sentences were presented in an auditory condition, and it was ensured that these sentences were different to those used in the behavioral assessment of speech understanding. The duration of the blocks ranged from 12 to 14 s depending on the specific sentences in the block. The levels of all the sentences were normalized to the same root mean square level. The corresponding visual condition consisted of silent videos of a female talker speaking IEEE sentences from the same corpus (Fig. 1B).

### fNIRS data acquisition

Testing was performed in a sound-treated booth. Cortical responses were measured using the Hitachi ETG-4000 continuous-wave fNIRS system (Hitachi Medical Co., Japan). A 3 × 11 grid array containing 52 channels (source-detector pairs) was used, with an inter-optode separation of 30 mm. The array was positioned over the participants’ temporal and occipital lobes, in order to cover auditory and visual cortical areas (see Fig. 1C). This placement was guided by the International 10-20 system (Jasper 1958) and careful measurement of distance from nasion to inion and transverse distance from left preauricular point to right preauricular point, to maintain consistency between test sessions and participants. Specifically, the first sources at the left and right extremities of the bottom row of sources and detectors were aligned with the left and right pre-auricular points, respectively (T7 and T8 relative in terms of 10-20 reference points) and the source at the midpoint of the array row when positioned over the occipital lobe was aligned to Oz (Jasper 1958).

### Anatomical guidance and defining regions of interest for correlation analysis

Regions of interest (ROIs) were defined for the left and right superior temporal gyri (STG) separately, and left and right occipital gyri, corresponding to auditory and visual cortical areas, respectively. Studies by Shader et al. (2021) and Chen et al. (2015) similarly examined cortical activity in both auditory and visual areas using fNIRS and demonstrated area specificity of activations, whereby auditory areas responded largely to auditory stimuli and visual areas to visual stimuli.

Subject-specific anatomical landmarks and optode positions were recorded using an electromagnetic 3D Polhemus Probe Positioning Unit (Vermont, USA; http://www.polhemus.com). This digitized, subject-specific information was later registered to the Colin27 atlas, using the AtlasViewer toolbox (Aasted et al. 2015), which performs a transformation to register an anatomical model to the optode locations. The optode locations were then projected towards the cortical surface of the registered atlas model to provide an estimation of the cortical Montreal Neurological Institute coordinates associated with an optode pair (or channel). Given that the Colin27 atlas is parcellated, these projections were used to provide relevant cortical labels for the region to which a channel is likely sensitive.

In order to visualize the fNIRS results on a cortical surface, forward modeling was conducted within AtlasViewer using the built-in Monte Carlo photon transport package. This process provides an estimate of the sensitivity of the fNIRS measurement to regional changes in chromophore concentration, specifically the probability of the detected NIR light traveling through a given region of tissue. A Monte Carlo simulation (100,000 photons) was run to produce a model of where the detected photons will have traveled. Measurement sensitivity profiles generated from the Monte Carlo simulations were then used to perform image reconstruction using custom MATLAB scripts, in order to visualize results.

### Processing and statistical analyses of fNIRS data for channel-wise cortical activation maps

The NIRS Brain AnalyzIR Toolbox (Santosa et al. 2018) was used to preprocess and analyze the fNIRS data. Raw light intensity was resampled to 5 Hz, before being converted to optical density. Levels of oxygenated (HbO) and deoxygenated (HbR) hemoglobin concentration were then determined using the modified Beer–Lambert law. To account for serially correlated errors such as physiological noise, e.g. heart rate, respiration, and Mayer waves (Yücel et al. 2016; Luke, Larson, et al. 2021a), data were pre-whitened as per Barker et al. (2013). The first-level analysis was conducted using the iteratively reweighted least-squares (AR-IRLS) method, employing the default toolbox canonical hemodynamic response function (HRF), convolved with a boxcar model, which specified the duration of stimulus events. This analysis was performed across all channels for each subject, which computed an estimated regression coefficient (beta) for each channel in the array, representing the absolute estimated “strength” of hemodynamic activity for each condition. Group-level analysis was performed using a linear mixed model to examine the interaction of experiment group (CI vs. NH), stimulus (speech vs. non-speech), and condition (visual vs. auditory) (fixed effects), with each subject treated as a random effect. A linear mixed modeling approach was adopted for all group-level analyses of the fNIRS data. This is able to account for variance associated with differences across individuals through specification of random effects and is robust in cases where data are missing or unbalanced across groups (Boisgontier and Cheval 2016).

A time-series analysis was initially conducted to qualitatively inspect grand average time courses of the fNIRS responses (examples shown in Fig. 3A) across source-detector pairs for channels selected for ROIs covering auditory and visual areas; however, this approach was not used in the quantitative statistical analysis using the linear mixed model. For the qualitative analysis, raw light intensity from the channels of interest was first converted to optical density. The temporal derivative distribution repair (TDDR) procedure was applied to the data to account for motion artifacts, removing baseline shift and spike artifacts (Fishburn et al. 2019). HbO concentration was then determined using the modified Beer–Lambert law. A bandpass filter of 0.01–0.3 Hz was applied. The data were then epoched into 5 s windows prior to and 30 s post-stimulus onset.

We first examine expected “intra-modal” activations in the auditory and visual modalities in key ROIs covered by our fNIRS array (namely, auditory and visual cortices). Brain activations were quantified using the beta values for both HbO and HbR computed in the linear mixed model. For each channel, we first verified that the changes in HbO and HbR concentration in response to each stimulus and condition did not significantly differ from the canonical model, i.e. HbO was observed to be increasing and HbR decreasing. These beta values were then further subjected to the multivariate Hotelling’s *T*^2^ test (Santosa et al. 2018) to take account of the joint behavior of HbO and HbR and determine whether both are significantly different from baseline at *q* < 0.05 (corrected *P* value for multiple comparisons, using the Benjamini–Hochberg procedure; Benjamini and Hochberg 1995). While this measure of joint activity of HbO and HbR was not used in later analyses, e.g. correlations with behavioral measures, this was an important initial step in our fNIRS analysis to confirm and characterize activations across all groups (NH and CI), stimuli (speech and non-speech), and conditions (visual and auditory).

In order to examine the hypothesis of a stimulus-specific effect for visual stimuli and resultant “cross-modal” activation patterns for both groups, a separate linear mixed effects model was conducted on data from the visual condition only. The model examined the interaction of group and stimulus as fixed effects, and each subject was again treated as a random effect. For channels showing a significant group and stimulus type interaction, post hoc *t*-test contrasts were computed for HbO and HbR separately with Benjamini–Hochberg correction for multiple comparisons (Benjamini and Hochberg 1995), to address (i) whether for each level of group (CI and NH), there was a significant difference in activated regions in response to speech versus non-speech stimuli and (ii) whether for each level of stimulus type (speech and non-speech), there was a significant difference in activated regions between groups (CI vs. NH).

To extract a single estimate of activity per ROI, a weighted averaging of individual beta values was performed across channels within each ROI, where weights were equivalent to the inverse of the standard error of the GLM fit for each channel (Santosa et al. 2018). This computed a single beta value quantifying intra-modal or cross-modal activity for each ROI, which was to be used in the correlation analysis with behavioral measures of speech understanding.

### Functional connectivity analysis

The metric of coherence was used to examine task-related interactions between auditory and visual ROIs (Sun et al. 2004; Bowyer 2016) and has been successfully employed in studies seeking to explore stimulus-dependent temporal dynamics of BOLD signals in both fMRI (e.g. Müller et al. 2001; Müller et al. 2003; Curtis et al. 2005) and fNIRS applications (e.g. Park et al. 2019). The MNE toolbox was used to process the data for the functional connectivity analyses (Gramfort et al. 2013, 2014; Luke, Shader, et al. 2021b). The channel selections for ROIs were the same as those used for the ROI analysis of cortical activations, consisting of left and right auditory and visual areas (see Fig. 1D). The connectivity analysis was performed on data HbO, as it has been shown to yield more robust coherence patterns and connectivity compared to HbR (Wolf et al. 2011). In the CI users, due to variations in coil positioning, some optodes located above the coil were not able to obtain fNIRS data, and thus, affected channels were excluded from the analysis. Specifically, 2 out of 3 channels were affected in the left auditory ROI for 3 subjects, and up to 2 out of 3 channels were affected for only 2 subjects in the right auditory ROI. No channels needed to be excluded for the visual ROIs.

Raw light intensity from these channels of interest was first converted to optical density and TDDR was applied to the signal. HbO concentration was then determined using the modified Beer–Lambert law. A bandpass filter of 0.01–0.3 Hz was applied. The data were then epoched to a time window during which an auditory or visual stimulus was being presented and a response occurred (−23 to +37 s). Coherence was calculated using epoched data in the specified frequency range of 0.05–0.3 Hz between all channels for each subject (Tong et al. 2011). The resultant coherence values represent the similarity in signal between each channel pair in the specified time window, with values closer to 1 indicating greater similarity between the 2 signals and values closer to 0 indicating greater independency of the signals (Sun et al. 2004). These values were then exported for processing in R (R Core Team 2021) with the *lme4* package (Bates et al. 2015) to perform separate linear mixed effects models for each ROI pair between the 4 ROIs—Left Auditory–Left Visual; Right Auditory–Right Visual; Left Auditory–Right Visual; Right Auditory–Left Visual; Left Auditory–Right Auditory; and Left Visual–Right Visual, examined separately according to condition (auditory or visual). A linear mixed effects modeling approach was again used, as this accounts for the multiple data points within individuals, therefore although observations were removed for missing values—for example, where coil placement interfered with channel activity—the remaining responses within that subject could still be accounted for in the analysis (Boisgontier and Cheval 2016; Brown 2021). Coherence values between ROI pairs were the dependent variable in the model, with fixed effects of group and stimulus type and their combined interaction. “Subject” was included as a random effect to account for potential variation given the multiple measurements per subject. Side of stimulation was included as a fixed effect in the models for responses to the auditory condition. Significance was calculated using the *lmerTest* package (Kuznetsova et al. 2017), which applies Satterthwaite’s method to estimate degrees of freedom and calculates *P*-values for mixed models. The model specification was “beta (coherence values) ~ Group^*^Stimuli + (1| Subject ID)” for the visual condition and “beta (coherence values) ~ Group^*^Stimuli + Stimulation side + (1| Subject ID)” for the auditory condition. Post hoc comparisons were then computed using the *emmeans* package (Lenth 2020), to further examine significant interaction, which provided significance of pairwise contrasts of estimated marginal means for fixed effects group and stimulus, e.g. “CI speech – NH speech,” with applied Benjamini–Hochberg adjustment.

### Correlation analysis of brain activity and behavioral measures

Correlation analyses were conducted to examine relationships between amplitude-based and connectivity measures of cross-modal activity and behavioral speech recognition performance. For connectivity measures, in order to examine the relationship between interregional connectivity and behavioral measures of speech understanding, coherence values of single “seed” channel pairs within the left auditory, right auditory, left occipital, and right occipital ROIs were selected based on the strongest peak responses observed at the group level in the auditory area to auditory stimuli and in the visual area to visual stimuli (both speech and non-speech, see Fig. 1D for channel selections).

A nonparametric bootstrap approach (2,000 samples) was used to obtain confidence interval estimates (bias-corrected, “BCa”) of Spearman’s *r* and test the null hypothesis that there was no association between HbO activation (beta weight) for amplitude-based measures or task-related coherence value for each seed channel pair for connectivity measures, to either a speech or non-speech stimuli in an auditory or visual condition in each of the ROIs, and behavioral speech recognition scores (specifically, auditory-only-in-noise, visual-only and “visual enhancement” (auditory-only score subtracted from the AV score)). The same approach was used to investigate the relationship between task-related coherence values of seed channels pairs and behavioral speech recognition scores, again using Spearman’s rank-order correlation.

## Results

### CI users show enhanced lipreading abilities and benefit from visual cues in speech processing tasks

**Fig. 2:**
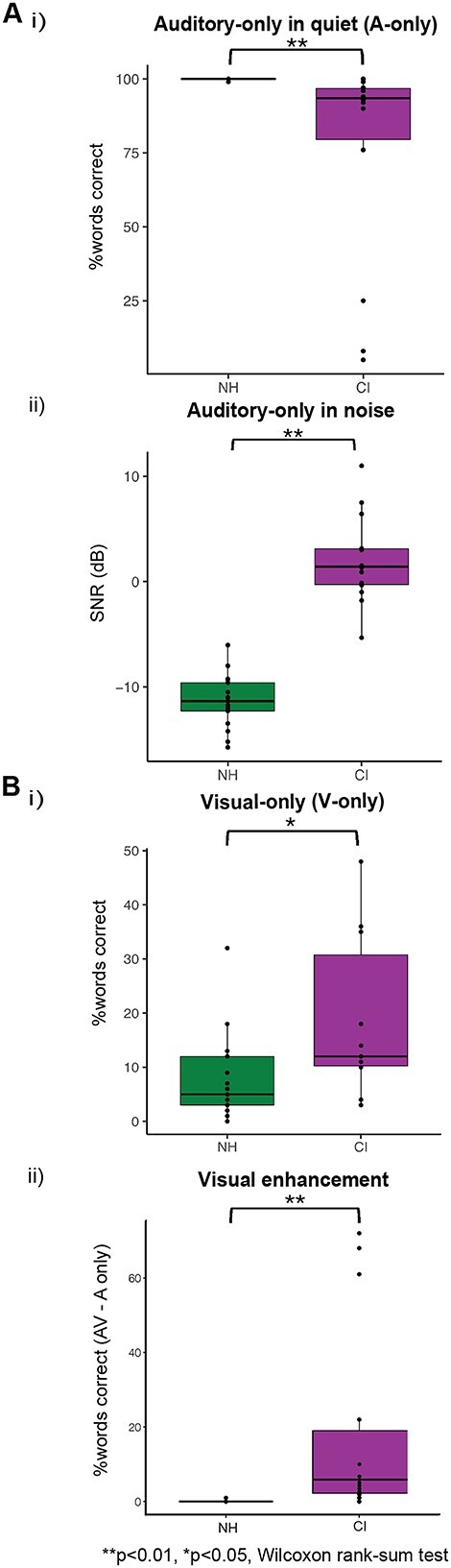
Behavioral speech recognition measures. A) Boxplots show distributions of scores for speech understanding in an auditory-only condition (IEEE sentences), (i) in quiet and (ii) in noise (CCRM task), where “SNR” represents the signal-to-noise ratio at which the participant achieved 50% correct (lower score = better performance). B) (i) Boxplots show speech understanding in a visual-only condition (IEEE sentences) for both NH and CI groups and (ii) “visual enhancement,” calculated as the difference between performance on the AV condition and performance on the auditory-only condition.

Visual cues provide one means with which to assess language processing (Capek et al. 2010), particularly in deaf and hard of hearing individuals—including CI users—for whom auditory input may be substantially degraded (Peelle and Sommers 2015). To determine the extent to which CI users exploit visual cues for speech understanding, we assessed their ability to understand speech using auditory or visual cues only, or both combined, and compared these abilities to those of a group of age-matched, NH listeners. Listeners in both groups were presented with single sentences (selected from the IEEE corpus) and were instructed to repeat as many words as they were able to identify, a task we scored in terms of “whole words correct.” Speech-in-noise performance was also assessed using a Co-ordinate Response Measure (CRM) paradigm (Bolia et al. 2000; Brungart 2001). As expected, NH listeners were able to recognize speech with auditory-only cues significantly better than CI users, both in quiet (*W* = 21.5, *P* < 0.001, *r* = 0.75) and in the presence of background noise (*W* = 238, *P* < 0.001, *r* = 0.84; Fig. 2A, i) and ii)). In contrast, CI users were more accurate than NH listeners in recognizing IEEE sentences presented exclusively visually (*W* = 85, *P* = 0.010, *r* = 0.47; Fig. 2B, i). Both NH and CI groups reached ceiling performance in the AV condition, and a “visual enhancement” metric—the difference between the speech-in-quiet score subtracted from the AV score—to quantify the degree of benefit obtained from visual cues when both auditory and visual speech information were presented. Due to their already high performance in the auditory-only task, NH listeners showed minimal need for visual cues and therefore demonstrated no visual enhancement. On average, CI users showed comparatively higher visual enhancement (*W* = 217, *P* < 0.001, *r* = 0.75; Fig. 2B, ii), and this varied between listeners. This variability in performance between CI users provides a potential means with which mechanisms underlying language processing might be assessed.

**Fig. 3:**
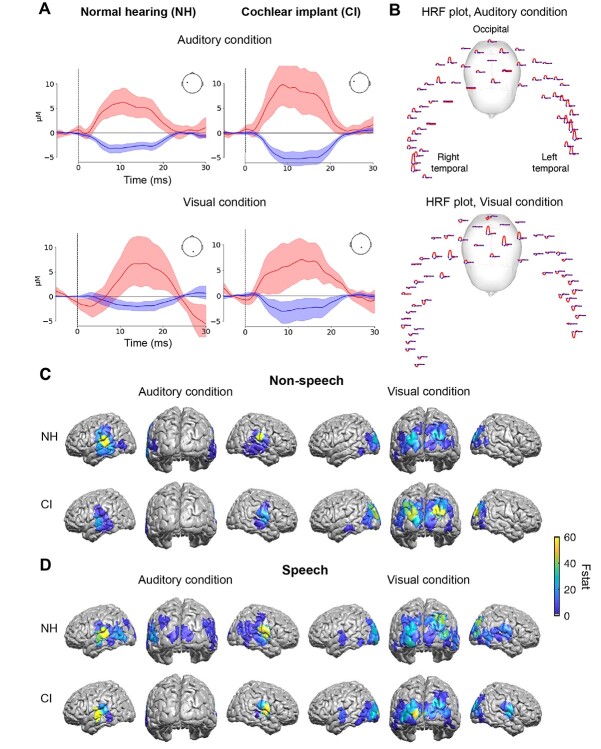
Channel-wise analysis of neural activation patterns in NH and CI groups. A) Grand-average time courses, processed for initial qualitative inspection, from a single source-detector pair (channel) in the left auditory (top) and visual ROIs (bottom) showing HbO and HbR hemoglobin responses to auditory and visual speech stimuli from both NH and CI groups. B) Example of canonical HRFs across the optode array in response to auditory stimuli (top) and visual stimuli (bottom) following group-level analysis and canonical model fit. C) Projection of *F*-statistic (*F*stat) maps to cortex following group-level analysis, showing significant Hotelling’s *T*^2^ test results, quantifying the joint activity of oxy- and deoxy- hemoglobin (HbO and HbR, respectively) for both NH and CI participants (corrected *P* value <0.05) in response to non-speech stimuli in both auditory and visual conditions and D) *F*stat maps in response to speech stimuli in both auditory and visual conditions.

### Intra-modal and cross-modal auditory and visual cortical activity differ for speech and non-speech stimuli

Speech processing is thought to utilize common neural pathways—predominantly in the left hemisphere between temporal, parietal, and occipital regions—regardless of presentation modality (Bernstein and Liebenthal 2014; Peelle et al. 2022). As such, we examined the extent to which auditory and visual speech and non-speech stimuli generate intra-modal and cross-modal cortical activity in both NH listeners and CI users, using fNIRS to estimate changes in cortical hemoglobin concentrations and, from this, inferring changes in regional neural activity specific to language content.

To confirm expected responses in auditory and visual cortical areas, we assessed “intra-modal” activations—defined by activations to auditory stimuli in auditory (temporal) areas and to visual stimuli in visual (occipital) areas—within key ROIs in auditory and visual cortices. Non-speech stimuli comprised amplitude-modulated (AM) noise and concentric gratings in the auditory and visual modalities, respectively; speech stimuli were IEEE sentences (Rothauser et al. 1969) presented in auditory-only and visual-only modalities. Superior temporal regions were bilaterally activated in both NH listeners and CI users (Fig. 3C and D, auditory condition), consistent with fMRI responses to AM noise (Giraud et al. 2000) and speech stimuli (Adank 2012). Also consistent with evidence from EEG and fMRI analyses, respectively, visual stimuli—both non-speech (Doucet et al. 2006) and speech (Calvert et al. 1997)—activated occipital regions in NH and CI participants (Fig. 3C and D, visual condition).

Cross-modal cortical activity, proposed to represent recruitment of sensory-deprived regions by other “intact” sensory systems, was assessed to address the hypothesis that language-based stimuli such as speech might naturally recruit regions cross-modally as part of a multimodal language network. We therefore measured fNIRS activation to visual stimuli in auditory areas, and activation to auditory stimuli in visual areas. Evidence of cross-modal activity was observed in both NH and CI participants (Fig. 3C and D). Notably, cross-modal activation of superior temporal regions to visual speech was evident in both NH and CI groups (Fig. 3D, visual condition), and this was confirmed in the whole group analysis as well as in a subset of CI participants receiving right-side auditory stimulation only, the latter assessed to account for the heterogeneity in terms of the ear implanted. Controlling for side of stimulation in this way did not influence the outcome—significant cross-modal activation of superior temporal regions to visual speech was present in (right-implanted) CI users with right-side auditory stimulation (see Supplementary Fig. 3). This cross-modal activity in auditory cortex to visual stimulation concords with existing fMRI evidence indicating temporal activity in response to visual speech in both deaf and hearing subjects (Calvert et al. 1997; MacSweeney et al. 2002; Hall et al. 2005). Together with the observed intramodal activation to auditory speech, this is consistent with a role for auditory cortical regions in speech processing, independent of hearing acuity, or the modality—auditory or visual—by which speech is conveyed.

**Fig. 4:**
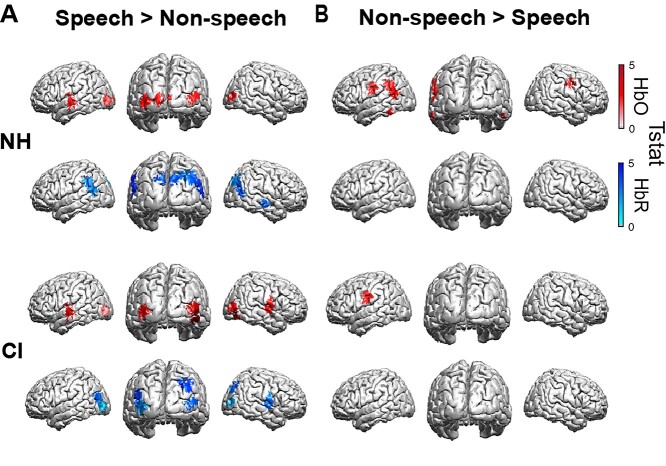
Significant channel-wise differences in activation to visual stimuli for speech contrasted with non-speech. Statistically significant activations following post hoc *t*-test contrasts on channels showing a significant interaction of group and stimulus type (see Supplementary Table 1). Differential activations are shown for both chromaphores HbO (red) and HbR (blue) “within” group (CI and NH) for each stimulus type (speech vs. non-speech). A) Speech > Non-speech and B) Non-speech > Speech.

We hypothesized that speech and non-speech stimuli engage different functional cortical networks, which may be reflected in differential recruitment of specific brain regions depending on the stimulus type, and, specifically, that visual speech engages auditory cortex (see fMRI studies by: Calvert et al. 1997; Hall et al. 2005; MacSweeney et al. 2002). To test this hypothesis, we conducted an analysis on data from the visual condition only, in order to examine the specific interaction of group and stimulus type. Post hoc *t* test contrasts were then computed for oxygenated and deoxygenated hemoglobin (HbO and HbR, respectively) recorded by optical channels showing a significant group × stimulus interaction (see Supplementary Table 1), to investigate the existence of significant differential interaction in a contrast of responses to speech versus non-speech visual stimuli within each group. Consistent with our hypothesis, left superior temporal gyrus (STG) showed greater activation to visual speech when contrasted with non-speech stimuli in both NH [*t*(58) = 2.304, corrected *P* = 0.029] and CI [*t*(58) = 3.267, corrected *P* = 0.007] subjects (Fig. 4A). We also assessed, for each stimulus type, any significant differences in activated regions between groups (CI vs. NH) and found that activation of superior temporal regions to visual speech was not significantly different between NH listeners and CI users [(*t*(58) = 0.881, corrected *P* = 0.754)].

Together, our data demonstrate that auditory cortical areas may be activated by speech in either mode of presentation (auditory or visual) and that visual cross-modal activity in the auditory cortex depends on stimulus type (i.e. speech vs. non-speech) in CI users. Specifically, we observed greater activation of superior temporal regions to visual speech than to nonvisual speech, supporting the notion of a multimodal speech processing network that represents coordinated activity between different brain regions, e.g. auditory and visual, to support speech processing (Bernstein and Liebenthal 2014; Peelle et al. 2022).

### Enhanced cortical connectivity in CI users for speech compared to NH listeners

Having established the presence of distributed activation of cortical areas in response to speech versus non-speech stimuli, we explored the role for a hypothesized multimodal speech network by assessing task-related connectivity between 4 main cortical ROIs (left auditory, right auditory, left visual and right visual) in response to auditory and visual speech stimuli. Specifically, we assessed the level of coherence between the left and right temporal and left and right occipital ROIs to determine the degree of connectivity between them during speech processing tasks in both modalities. Separate linear mixed-effects models were computed for the coherence values of each ROI pair, per condition (auditory vs. visual) (see Tables 2 and 3 for a summary of estimates from the models).

**Table 2:** Estimates of coherence for each ROI pair, visual condition.

	Left Auditory–Left Visual	Left Auditory–Right Visual	Left Auditory–Right Auditory	Right Auditory–Right Visual	Right Auditory–Left Visual	Left Visual–Right Visual
	Estimate (SE)	Estimate (SE)	Estimate (SE)	Estimate (SE)	Estimate (SE)	Estimate (SE)
Intercept	0.61 (0.04)^a^	0.62 (0.04)^a^	0.67 (0.04)^a^	0.06 (0.04)^a^	0.59 (0.04)^a^	0.77 (0.03)^a^
Group(CI)	−0.05 (0.06)	−0.06 (0.06)	−0.06 (0.06)	−0.02 (0.06)	−0.01 (0.06)	−0.05 (0.05)
Stim(Speech)	−0.03 (0.01)	−0.05 (0.01)^a^	−0.03 (0.01)^c^	−0.02 (0.01)	0.01 (0.01)	−0.03 (0.01)
Group(CI): Stim(Speech)	0.06 (0.02)^c^	0.07 (0.02)^b^	0.00 (0.02)	0.00 (0.02)	−0.01 (0.02)	0.02 (0.02)

Model notation: ß ~ group ^*^ stimulus + (1| subject). Stim, stimulus (Speech or Non-Speech); Group(NH) and Stim(Non-Speech) are on the intercept.

Significance codes*:*  ^a^*P <* 0.001, ^b^*P <* 0.01, ^c^*P <* 0.05.

**Table 3:** Estimates of coherence for each ROI pair, auditory condition.

	Left Auditory–Left Visual	Left Auditory–Right Visual	Left Auditory–Right Auditory	Right Auditory–Right Visual	Right Auditory–Left Visual	Left Visual–Right Visual
	Estimate (SE)	Estimate (SE)	Estimate (SE)	Estimate (SE)	Estimate (SE)	Estimate (SE)
Intercept	0.61 (0.04)^a^	0.62 (0.04)^a^	0.66 (0.04)^a^	0.61 (0.04)^a^	0.59 (0.04)^a^	0.76 (0.03)^a^
Group(CI)	−0.07 (0.06)	−0.07 (0.06)	−0.08 (0.06)	−0.07 (0.06)	−0.05 (0.06)	−0.03 (0.05)
Stim(Speech)	−0.03 (0.01)	−0.05 (0.01)^a^	−0.02 (0.01)	−0.02 (0.01)	0.00 (0.01)	−0.01 (0.01)
Stimside(Left)	0.04 (0.05)	−0.03 (0.05)	−0.00 (0.05)	0.01 (0.05)	0.04 (0.05)	−0.06 (0.04)
Group(CI): Stim(Speech)	0.07 (0.02)^b^	0.08 (0.02)^a^	0.02 (0.02)	0.04 (0.02)	0.02 (0.02)	0.03 (0.02)

Model notation: ß ~ group ^*^ stimulus + side of stimulation + (1| subject). Stim, stimulus (Speech or Non-Speech); Stimside, side of auditory stimulation (Left or Right). Group(NH), Stim(Non-Speech), and Stimside(Right) are on the intercept.

Significance codes: ^a^*P <* 0.001, ^b^*P <* 0.01.

The fixed effects of group and stimulus type and their combined interaction were examined, with subject specified as a random effect. Side of stimulation was included as a fixed effect in the models for responses to the auditory condition. The models of particular interest were “Left Auditory–Left Visual” and “Left Auditory–Right Visual” in response to visual stimuli, with a data-driven hypothesis following observation of increased left auditory cortical activity to visual speech in the CI group, which was significantly associated with lipreading abilities in the CI users. We hypothesize that if this activation is representative of network activity supporting speech processing between auditory and visual regions in CI users, then they will show greater connectivity—i.e. higher coherence—for speech compared to non-speech stimuli.

For speech and non-speech visual stimuli, assessment of coherence between left auditory to left visual ROIs revealed a significant interaction of group and stimulus (ß = 0.07, SEM = 0.02, *P* = 0.01), with greater coherence to speech stimuli observed in CI users compared to NH listeners. This significant interaction of group and stimulus was also observed for the pairing of left auditory to right visual ROIs (ß = 0.07, SEM = 0.02, *P* = 0.002). For auditory stimuli, coherence between left auditory and left visual ROIs also showed a significant interaction between group and stimulus type (ß = 0.07, SEM = 0.02, *P* = 0.007), as did coherence between left auditory and right visual ROIs (ß = 0.08, SEM = 0.02, *P* = 0.001). No significant interaction of group and stimulus was observed in any of the other ROI pair models for either visual or auditory stimuli (see Tables 2 and 3 for model summaries). These data suggest a role for left auditory to visual ROI activity in differential processing of speech and non-speech stimuli in both visual and auditory modalities, consistent with our hypothesis, as a function of group—whereby CI users show greater coherence to speech stimuli compared to NH listeners.

**Fig. 5:**
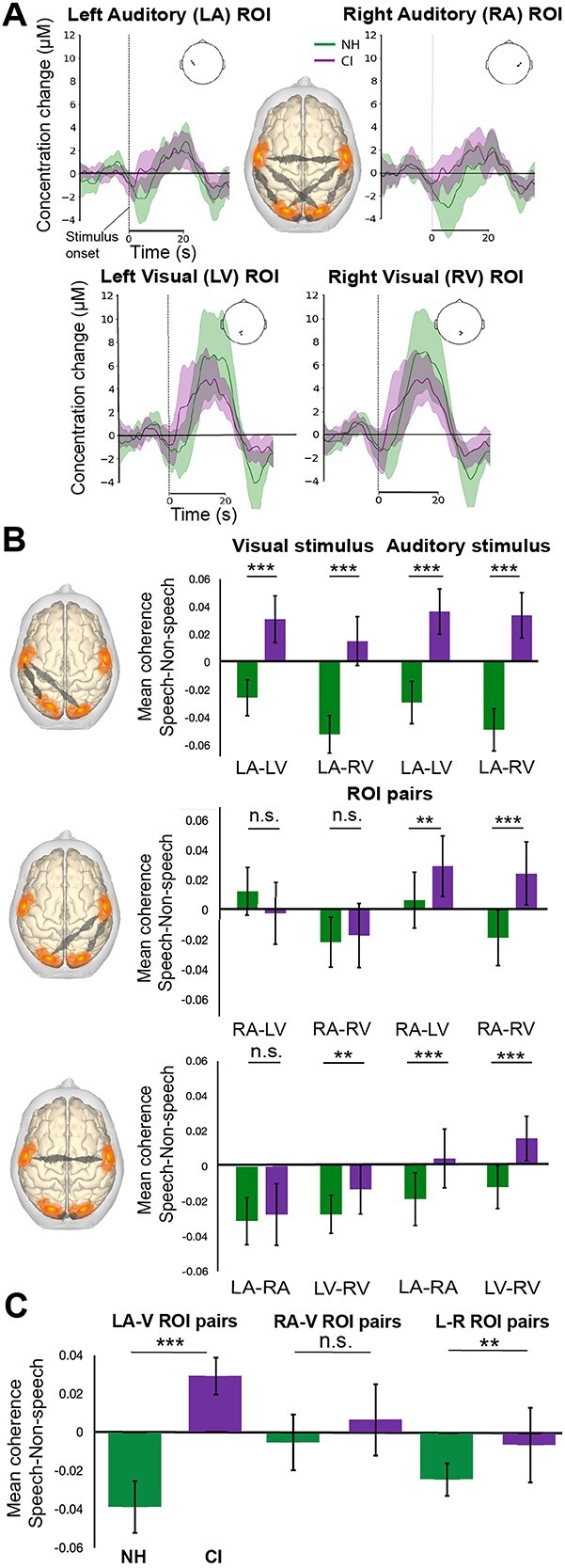
Task-related functional connectivity analysis. A) Schematic representation (brain at center) of functional connectivity analyses conducted between 4 main ROIs (left and right auditory, left and right visual) in response to speech and non-speech stimuli in visual-only and auditory-only conditions. Waveform plots show examples of the epoched time window where task-related responses to visual speech occurred within each main ROI for both CI and NH groups. B) Bar plots with standard deviation bars represent estimated marginal means of coherence in both visual (column Visual stimulus) and auditory (column Auditory stimulus) conditions for models (from top row: Left Auditory to Left Visual (LA–LV), Left Auditory to Right Visual (LA–RV); middle row: Right Auditory to Left Visual (RA–LV), Right Auditory to Right Visual (RA–RV); bottom row: Left Auditory to Right Auditory (LA–RA), Left Visual to Right Visual (LV–RV)), where a difference metric of interregional coherence to Speech – coherence to Non-Speech stimuli is presented. Therefore, a positive value indicates greater coherence to speech relative to non-speech, and vice versa. C) Bar plots with standard deviation bars showing overall mean difference of Speech – Non-Speech coherence, collapsed across both visual and auditory conditions, for (i) Left auditory to both left and right visual regions (combined); (ii) Right auditory to both left and right visual regions (combined); and (iii) Left to right hemisphere ROI pairs (i.e. LA-RA, LV-RV). ^***^Statistically significant relationship (*P* < 0.001), ^**^(*P* < 0.01).

To investigate further the main hypothesis relating to stimulus-specific coherence in CI users, i.e. that cross-modal activation may differ with stimulus type, reflecting engagement of a network supporting speech processing, we examined post hoc comparisons of estimated marginal means—specifically, the difference in mean coherence in response to speech stimuli relative to non-speech stimuli within NH listeners and CI users and for each ROI pair (see Fig. 5B). Whereas NH listeners showed reduced coherence between left auditory area and visual areas for speech compared to non-speech stimuli (*M* = −0.04, SD = 0.01), the opposite was true for CI users, who showed greater coherence to speech stimuli relative to non-speech stimuli (*M* = 0.03, SD = 0.01) across both visual and auditory conditions. This difference in coherence to speech compared non-speech stimuli was statistically significant between the groups [*t*(27.90) = 16.15, *P* < 0.0001] (see Fig. 5B (top row) and Fig. 5C).

For the right auditory cortical region, significant differences in coherence to speech relative to non-speech stimuli between NH and CI groups were evident only for auditory stimuli, and the overall difference averaged across visual and auditory conditions was not significantly different between the 2 groups [*t*(22.26) = 1.872, *P* = 0.07] (see Fig. 5B (middle row) and Fig. 5C). The mean coherence “difference” values for speech – non-speech show greater divergence between NH and CI groups for left auditory cortex to visual cortex ROI pairs (Fig. 5B and C). While this pattern of divergence between groups was less evident for right auditory to visual cortex ROI pairs, this activity appears to be correlated between left and right hemispheres—suggestive of stronger interregional network activity driven by left auditory cortex, which is present, but reduced, in the right hemisphere.

For the interhemispheric comparisons of Left Auditory–Right Auditory and Left Visual to Right Visual ROI pairs (Fig. 5B (bottom row) and Fig. 5C) both CI and NH subjects showed reduced coherence to speech relative to non-speech stimuli in the visual-only condition, however again diverged in the auditory-only condition, whereby CI users showed positive coherence to speech relative to non-speech stimuli. The overall mean difference in coherence for speech relative to non-speech stimuli between hemispheres, averaged across both visual and auditory conditions, was significant between the groups [*t*(15.52) = 3.114, *P* = 0.007].

Together, our data suggest a fundamental difference in the connectivity of the Left Auditory to Visual ROIs for CI users relative to NH listeners. Specifically, the difference in coherence in response to speech stimuli relative to non-speech stimuli is greater in CI users relative to NH listeners. This indicates increased use of the proposed language network (Peelle et al. 2022; Sherafati et al. 2022) for CI users when processing speech stimuli relative to the NH listeners, which might reflect the increased processing required to parse linguistic content for this group.

**Fig. 6:**
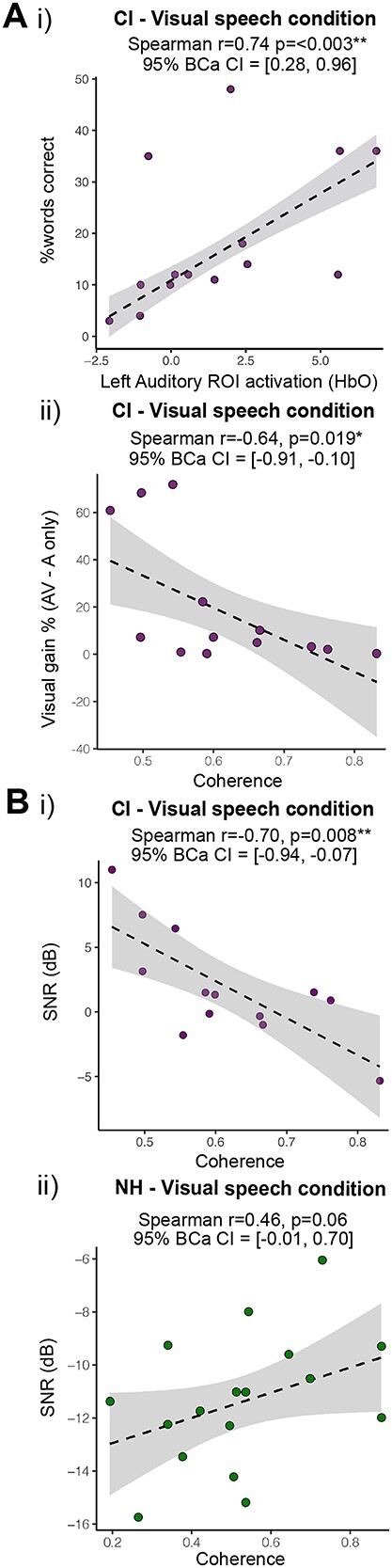
Correlation analyses between brain activity and behavioral speech recognition measures. Data are plotted with robust regression line and standard error confidence bounds. Confidence interval estimates (bias-corrected, “BCa”) of Spearman’s *r* are reported, determined from a bootstrap approach using 2,000 samples. A) (i) Relationship between cross-modal activity to visual speech and lipreading abilities in CI participants, indicating a positive association between performance on a visual speech (lipreading) task and HbO activation in left STG region; (ii) relationship with task-related coherence to visual speech between auditory and visual seed channel pairs and “visual enhancement,” representative of AV score—auditory-only score, indicating a negative association between visual enhancement score and degree of AV coherence. B) Relationships with coherence between seed channel pair (left auditory–left visual) in response to visual speech for (i) CI group versus (ii) NH group, and participants’ speech-in-noise abilities (where lower score indicates better performance). ^**^Statistically significant relationship (*P* < 0.01), ^*^(*P* < 0.05).

### Better lipreading abilities associated with greater activation of left auditory cortex in CI users

The variation in performance between CI users in tasks requiring lipreading or understanding speech in background noise afforded us the opportunity to explore potential contributing factors underlying processing of language-based stimuli and the role of cross-modal activations in these tasks. To investigate the relationship between amplitude of fNIRS responses and behavioral performance in lipreading and in speech understanding in noise, cross-modal activity was quantified using the “beta” value representing HbO amplitude of activation to visual stimuli in auditory ROIs (corresponding to left and right STG), and activation to auditory stimuli in visual cortex. Specifically, a correlation analysis was conducted to examine relationships between cross-modal activity in auditory brain areas to visual stimuli and cross-modal activity in visual area to auditory stimuli, and measures of speech understanding in auditory-only and visual-only conditions, and the metric of “visual enhancement” (speech understanding performance in an auditory-only in quiet task subtracted from performance in an AV task). No statistically significant correlations were observed between the amplitude of cross-modal activation to speech or non-speech stimuli and auditory-only speech performance, in quiet or in noise in CI users. The fact that many of the CI users were high performers might be a contributing factor—most achieved >90% performance in the “auditory-only in quiet” paradigm (Fig. 2A, i). Although the CI cohort also showed a larger range of speech-in-noise abilities (Fig. 2A, ii; “auditory-only in noise”), no significant correlations were observed between the amplitude of cross-modal activation in auditory or visual regions and behavioral performance in the auditory speech-in-noise condition. Further, there were no significant correlations between the amplitudes of cross-modal activation and the difference in speech understanding between AV and auditory-only speech conditions (visual enhancement) (Fig. 2B, ii).

In contrast to the auditory speech stimuli, however, a significant correlation was observed between the amplitude of cross-modal auditory cortical activation to visual speech and lipreading abilities in CI users (Fig. 6A, i). Bootstrapped confidence interval estimates for Spearman’s *r* (*r*_s_) (based on 2,000 samples) indicated a significant positive relationship between performance on a visual speech (lipreading) task and HbO activation in CI users in the left auditory ROI, corresponding to STG (*r*_s_ = 0.74, 95% BCa CI = [0.28, 0.96], *P* = 0.003). This correlation remained significant and in the same, positive direction even with left-stimulated CI subjects removed for the analysis (Spearman *r* = 0.87, 95% BCa CI = [0.25, 1.00], *P* = 0.003), confirming that this relationship was not a result of heterogeneity in the side of stimulation within the CI group. This positive correlation between cross-modal activation to visual speech and lipreading ability supports the involvement of auditory cortical regions in speech processing, regardless of the modality of the presented speech.

### Greater activation of language network to visual speech in CI users is associated with better understanding of auditory speech-in-noise

Employing a contrast between speech and non-speech stimuli, designed specifically to assess the specificity of any cross-modal activation in language processing, we have demonstrated that connectivity between left auditory and occipital ROIs is greater in CI users relative to NH listeners, suggesting increased distributed neural resource requirements for this group which, overall, shows relatively poor performance in speech-in-noise tests compared to NH listeners. To determine whether this hypothesized language network, more strongly engaged for speech stimuli compared to NH listeners, influences performance in CI users, we examined the relationships between functional connectivity of ROI pairs in response to cross-modal stimuli, and speech understanding in auditory-only and visual-only conditions and found the strength of connectivity, assessed in terms of coherence, to be a significant factor in explaining performance. Specifically, Spearman’s correlation analysis revealed a significant relationship in CI users between understanding auditory speech-in-noise (where lower scores mean better performance) and increased coherence between left auditory and visual seed channels to visual speech (*r*_s_ = −0.70, 95% BCa CI = [−0.94, −0.07], *P* = 0.008) (Fig. 6B, i). The same relationship for understanding auditory speech-in-noise was not statistically significant for coherence values between the same 2 regions for visual speech stimuli in the NH group (*r*_s_ = 0.46, 95% BCa CI = [−0.01, 0.80], *P* = 0.06) (Fig. 6B ii).

We also examined the relationship between the metric of visual enhancement (i.e. the difference in speech understanding between auditory only and AV conditions) and connectivity of the putative language network. A significant relationship was observed between task-related coherence of left auditory and visual seed channels to visual speech and the metric of “visual enhancement” in CI users, whereby subjects with greater “visual enhancement” showed lower coherence values in response to visual speech (*r*_s_ = −0.64, 95% BCa CI = [−0.91, −0.10], *P* = 0.019) (Fig. 6A, ii). No significant correlations were observed between task-related coherence of any seed channel pair and relationship with visual-only measures.

Overall, our data suggest that enhanced coherence of activity between auditory and visual cortices in CI users in response to visual speech stimuli is positively correlated with the ability to understand auditory speech-in-noise. This relationship was not evident in NH listeners. Conversely, CI users who demonstrated the most performance “enhancement” from visual cues in an AV condition (i.e. they showed larger benefit in the AV vs. the auditory-only condition) showed lower connectivity between auditory and visual areas in response to visual speech. This is consistent with CI users with greater capacity for processing visual speech requiring fewer neural resources to process the linguistic content of speech.

## Discussion

We assessed task-related differences in cross-modal brain activity in auditory and visual cortical areas, in a cohort of postlingually deaf CI users and age-matched, NH listeners. We found that speech stimuli activated cortical regions within a distributed, multimodal network for speech processing and that the extent to which this putative language network is engaged by individual CI users is significantly related to their ability to understand speech in background noise. Specifically, greater coherence between the left auditory and visual cortices was associated with better speech-in-noise perception in CI users. Our data support the conclusion that cross-modal manifestations in auditory cortices of postlingually deaf CI users reflect “adaptive” activity of a speech network, recruited to support speech understanding. Reduced capacity to harness this network might lead to poorer listening performance in CI users.

### Cross-modal activation of auditory cortex is associated with improved perception of visual speech

Cross-modal plasticity has been proposed to limit auditory processing by occupying “higher-order” auditory areas with other sensory functions, e.g. visual-linguistic or related to visual motion, and hence may complicate the re-use of auditory functions (Kral 2007). Studies of cross-modal plasticity in CI users to date have typically sought to examine discrete, “functionally specialized” regions of auditory or visual cortex and measure the magnitude of responsiveness to stimuli from a different modality, e.g. activation to visual checkerboards in typically “auditory” superior temporal regions. These studies operated under the assumption that the relative magnitude of this activity in response to a visual stimulus reflects potentially “mal-adaptive” plastic changes due to sensory deprivation, or “adaptive” changes post-intervention (Stropahl et al. 2017). However, even NH individuals have been shown to engage left temporal regions during visual speech tasks (Calvert et al. 1997; MacSweeney et al. 2002; Hall et al. 2005). Here, we argue that differences in cross-modal influences, particularly when examined cross-sectionally, might be explained in part by the hierarchical nature of speech processing, including its manifestation at the cortical level in terms of a holistic representation of communication and language, rather than solely a series of acoustic events.

We observed differential activations in auditory cortical regions to visual speech compared to visual non-speech stimuli in NH listeners, as well as in postlingually deaf adults who use CIs. This suggests that cross-modal cortical activity in postlingually deaf CI users can be stimulus-specific and that, rather than the consequence of maladaptation to sensory deprivation, may reflect the distributed, multimodal nature of speech processing in the cortex. This conclusion is supported by a significant association between cross-modal activity to visual speech in left auditory regions and lipreading abilities in CI users, consistent with their better performance in lipreading compared to NH participants. Further, task-related connectivity between auditory and visual cortical areas in response to the speech stimuli—assessed to investigate this hypothesized language network—revealed a trend of enhanced cortical connectivity in CI users in response to both visual and auditory speech relative to non-speech stimuli. Together, these data suggest that cross-modal activity to visual stimuli in auditory areas is not necessarily detrimental to speech understanding in postlingually deaf CI participants but could reflect activation of multimodal cortical networks that contribute to speech processing, particularly where language-based stimuli are used. An important implication of this finding is that the choice of stimuli used to evaluate cross-modal plasticity in CI users is likely to influence any observed relationships with speech understanding, potentially a factor contributing to the variability in reported associations of cortical activity and performance outcomes (Stropahl et al. 2017).

### Understanding the variations in cross-modal activation by speech and non-speech stimuli

The degree of stimulus complexity (e.g. speech vs. non-speech) is a factor in determining which pathways in the brain’s speech and language network are activated or overlap with other networks. Historically, the lack of suitable neuroimaging approaches in CI users has made it difficult to disentangle these networks functionally—particularly when assessing activation magnitudes in single brain regions. For example, a negative correlation between auditory-speech understanding and cross-modal activity to low-level checkerboard stimuli has been reported using EEG in the right temporal areas of CI users (Sandmann et al. 2012; Kim et al. 2016); however, this relationship has not been consistently demonstrated across all studies (Buckley and Tobey 2011). The right superior temporal cortex has also been shown to respond to “low-level” visual stimuli in deaf individuals (Finney et al. 2001; Dewey and Hartley 2015), and it has been proposed that this cross-modal activity is a result of residual “sensory deprivation-induced” changes in cortical activity. However, from the perspective of a functioning cortical network, such activity in individuals with acquired deafness or even NH may also reflect activation of the ventral visual pathway (i.e. via the occipitotemporal cortex) supporting object recognition (Mishkin et al. 1983; Connor et al. 2007; Konen and Kastner 2008). The functional implications of this cross-modal activity to low-level visual inputs for auditory speech understanding are therefore still unclear, particularly as a significant negative correlation with auditory speech understanding in postlingually deaf CI users is not consistently reported (Buckley and Tobey 2011), including in our study.

Similarly, reports of cross-modal activity to visual speech and understanding spoken speech in postlingually deaf CI users are inconsistent in their findings. Zhou et al. (2018), for example, demonstrated greater cross-modal activation to visual speech in the left middle superior temporal lobe associated with poorer auditory speech understanding, proposing that this might arise from increased reliance on phonological processing and working memory, given a speculated role of the middle superior temporal lobe in a working memory network (evident in NH listeners; Vigneau et al. 2006; Zhou et al. 2018). Conversely, a positive correlation has been reported between increased cross-modal activity to visual sentences and auditory speech understanding, prior to, and 6 months after, cochlear implantation (Anderson, Wiggins, et al. 2017b). This result may reflect the restoration of speech envelope cues by visual speech, a mechanism that is more effective for “connected speech” such as sentences than single words or syllables (Peelle and Sommers 2015). Furthermore, performance in a visual speech perception or “speech-reading” task has also been positively correlated post-implantation with cross-modal activation of both auditory areas by visual speech stimulation (Rouger et al. 2012) and visual areas by auditory stimuli (Giraud et al. 2001). Consistent with these findings, our own observations of better lipreading performance in CI users with stronger cross-modal activation in the auditory cortex highlight the importance of interactive auditory and visual networks underpinning good speech comprehension, especially in AV situations.

### A multimodal and distributed language network can explain cross-modality in CI listeners

The multimodal interaction we observe in CI users with good lipreading and listening-in-noise abilities is consistent with evidence supporting a role for common pathways, relevant for speech processing in everyday communication, in both visual speech perception (Bernstein and Liebenthal 2014) and AV speech perception (Zion Golumbic et al. 2013; Crosse et al. 2015; Peelle and Sommers 2015; Karas et al. 2019). Functional MRI studies investigating the neural basis of “speech-reading” point to the existence of a distributed network of auditory and language regions, including the STG, posterior superior temporal sulcus (pSTS), premotor cortex, and the inferior frontal gyrus (Calvert et al. 1997; MacSweeney et al. 2000; Skipper et al. 2005; Okada and Hickok 2009; Okada et al. 2013). Such a multimodal speech processing network can explain why increased activation of temporal regions by visual speech stimuli has been linked with better auditory (Anderson, Wiggins, et al. 2017b) and visual language (Rouger et al. 2012) recognition in CI users. Underlying this improvement, cross-modal activity to “speech-reading” may reflect latent multimodal networks, regulated by multisensory experience preceding deafness (Lee et al. 2007). This is supported by increasing evidence highlighting the influence of visual information on auditory cortical activity, even in NH subjects (Bizley et al. 2007; Kayser et al. 2010; and see Crosse et al. 2015; Karas et al. 2019; Peelle and Sommers 2015; Zion Golumbic et al. 2013). Different, or multiple converging, mechanisms might underlie cross-modal activity in NH listeners relative to those relying on CIs in both visual and auditory speech tasks. However, the correlation between cross-modal activation of auditory cortex to visual speech and lipreading ability in CI participants (who were better overall performers in this task) (Fig. 6A, i), as well as between auditory–visual connectivity and auditory speech understanding (Fig. 6B, i), together suggests that speech understanding might be related to the engagement of a multimodal network supporting speech processing between auditory and visual cortical regions.

Evidence for an AV network and broader cross-regional synchronization in the processing of both visual-only and AV speech has been shown in an fMRI study with NH listeners (Peelle et al. 2022), including increased connectivity between visual cortex and a wide network of regions in temporal cortex and prefrontal cortex, supporting the conclusion that a “synergistic” relationship between auditory and visual networks is important for speech comprehension, particular in AV conditions (Anderson, Lazard, et al. 2017a). This may be particularly important for CI users given that activation in the left temporal lobe to visual speech increases immediately following implantation and can persist for up to 8 months (Rouger et al. 2012). Similar cooperation between left auditory and visual area observed in experienced CI users (Strelnikov et al. 2015) suggests that phonological processing and remapping of visual information onto auditory speech representations likely occur during adaptation to the CI device.

### Inter-regional cortical coherence as a measure of multimodal speech network engagement

The significant positive correlation we observed between connectivity values for left auditory and visual regions and performance in an auditory speech-in-noise task in CI users suggests a potential mechanism that benefits speech understanding in degraded conditions, relevant for the inherently multisensory nature of communication in everyday life. Certainly, information in visual speech stimuli can provide guidance through top-down mechanisms for auditory perceptual learning of vocoded acoustic signals and phonemic perceptual cues (Kral and Eggermont 2007; Ahissar et al. 2009; Bernstein et al. 2013) and, in general, provide critical cues for timing and content of an incoming acoustic signal (Peelle and Sommers 2015). It is possible, therefore, that cross-modal activations to visual speech may be related to mechanisms associated with AV speech processing that can be activated by visual inputs, even in the absence of auditory information.

Most importantly, connectivity between left auditory and visual cortical regions in response to visual speech was positively correlated with how well CI users were able to understand speech in background noise, and CI users who showed greater benefit from visual cues in an AV condition relative to auditory-only showed lower connectivity between auditory and visual regions. Notably, these effects were not observed for non-speech stimuli, suggesting a potentially enhanced AV mechanism in CI users to visual speech that is positively correlated with their auditory speech-in-noise understanding. This implies a functional benefit for such cortical connectivity under challenging listening conditions, which also appeared “weaker” for CI users who showed greater reliance on visual cues for speech understanding—suggesting that proficient lipreaders might require fewer neural resources when processing visual speech. One possible interpretation of enhanced auditory visual connectivity for speech stimuli is that it represents processes of AV integration, relevant for understanding speech in everyday listening tasks.

To date, few studies have examined task-related interregional connectivity in CI users. Chen et al. (2017) investigated cross-modal functional connectivity between auditory and visual cortices using fNIRS in a group of postlingually deaf CI users and age-matched NH controls. They reported, similar to the current study, that CI users exhibited increased cross-modal functional connectivity between visual and left auditory cortices for visual, as well as auditory, stimuli compared to NH controls and that differences between cross-modal functional connectivity for visual and auditory stimuli were correlated with speech-recognition outcomes in CI users, in that higher cross-modal connectivity for auditory than for visual stimuli was associated with better speech recognition abilities. However, an important difference between our study and Chen et al. (2017) is that they employed a nonlinguistic stimulus for the visual paradigm (i.e. flickering checkerboards) with an auditory paradigm consisting of a range of auditory stimuli such as tones, words, and reversed words. This inclusion of linguistic stimuli in the auditory paradigm and a nonlinguistic stimulus in the visual paradigm is a potential confound and limits ecological relevance to speech processing. Further, the auditory stimuli were presented concurrently with a silent video, which arguably engages directly a network for AV processing, and potentially explains the greater cortical connectivity they report for auditory stimulation.

Highlighting the importance not only of stimulus complexity but also the potential contribution of task-related factors when assessing cortical activity, Lazard and Giraud (2017) required their subjects to perform an active visual phonological processing task. They found evidence of right occipitotemporal cross-modal activity in their cohort of postlingually deaf CI users pre-implantation, which was negatively correlated with CI speech scores 6 months post-implantation. While an obvious difference with our study is their assessment of visual speech processing prior to implantation (with behavioral speech measures conducted 6 months post-op), their data suggest that connectivity between right auditory and visual areas is detrimental to recovery of listening function post-implantation; greater connectivity was associated with faster-than-average visual phonological processing in CI users with poor listening outcomes. They also observed that the left posterior STG/STS was activated in prospective CI users with good pre-implant lipreading scores, and this was associated with good speech outcomes post-implantation. Together, the data suggest that maintaining language-related processes in the left temporal region during deafness is important for good CI outcomes and that lipreading skills might play a role in preserving AV phonological processes critical to speech understanding post-implantation (Lazard and Giraud 2017).

### Limitations and replicability issues among CI studies of cortical activity and relationships with speech understanding

It is likely that different levels of stimulus complexity interact with different pathways in the brain’s speech and language network, or overlap with other networks, and that historical limitations in feasible neuroimaging approaches in CI users (e.g. poorer spatial resolution with techniques such as EEG) make it difficult to differentiate these networks functionally—particularly when assessed in terms of the magnitude of activation within a single brain region. A number of studies of postlingually deaf CI users have employed “low-level,” nonlinguistic stimuli such as concentric gratings (Doucet et al. 2006), checkerboards (Sandmann et al. 2012; Chen et al. 2016; Kim et al. 2016), and peripheral motion stimuli (Buckley and Tobey 2011) to assess cross-modal activity in auditory cortical regions. Although some report a negative correlation between cross-modal activity in right temporal cortex and auditory speech understanding (Sandmann et al. 2012; Kim et al. 2016) in response to checkboard stimuli (measured using EEG), this was not always the case. Buckley and Tobey (2011), for example, examined cross-modal activation in auditory cortex using peripheral motion stimuli and reported no significant correlations between cross-modal activity and auditory speech understanding in their group of 12 postlingually deaf CI users, although they found an association in a group of 10 prelingually deaf CI subjects. Chen et al. (2016) specifically investigated the influence of auditory and visual cortical “reorganization” (quantified using fNIRS responses to visual checkerboard stimuli in auditory and visual areas) on auditory speech understanding in a group of postlingually deaf CI users. Although they did not report a direct association between cross-modal activity in auditory area and speech understanding, they employed a combined “differential metric” to demonstrate elevated cross-modal activity to auditory input in visual cortex and reduced cross-modal activity to visual inputs in auditory cortex in CI users correlates with better speech performance.

Similarly inconsistent findings are reported in terms of relationships with cross-modal activity to visual speech in postlingually deaf CI users and speech understanding. It is possible that differences in the type of speech stimuli used, e.g. bisyllabic words in Zhou et al. (2018), compared to continuous speech in Anderson, Lazard, et al. (2017a), might explain ostensibly contradictory directions of correlation with measures of auditory speech understanding—perhaps related to the activation of different parts of a hierarchical language processing network. Notwithstanding these possible explanations for differences in outcomes reported, factors such as sample size, characteristics of CI subjects—age, duration of deafness, cognition, and etiology of deafness—as well as the specific stimuli employed and methodological approaches, particularly the metric used to correlate cortical activity with performance in listening tasks, all might contribute to the variations in reported outcomes.

Here, we suggest that cross-modal manifestations, like all cortical activations, are stimulus-dependent and may reflect activity of different functional networks. Activity in interregional brain networks should be accounted for in studies employing low-level, non-speech stimuli to examine cross-modal activity in temporal, “auditory” areas. Functional implications of cross-modal activity to low-level visual inputs for auditory speech understanding remain unclear—for example, it is not consistently reported that auditory speech understanding is correlated with cross-modal activity to low-level visual stimuli in postlingual CI users, with the potential for inherent variability in subject etiology or specific stimulus features employed influencing reported outcomes. So too, inferences about sites of cortical activation from scalp-based recordings (EEG) are not directly comparable to findings from functional imaging approaches, particularly in terms of the level of anatomical specificity possible using EEG (and fNIRS). Finally, longitudinal studies that provide multiple assessments post-implantation (potentially over years) may be required to determine those cortical processes that underlie adaptation from profound sensory loss to the process of sensory “restoration” with a CI. Ideally, such investigations would characterize at individual and population levels, controlling for key differences between pre- and postlingually deaf individuals to better understand the specific elements that contribute to the heterogeneity in outcomes. This would be facilitated by better characterization of cross-modal manifestations in nonsensory-deprived, “control” populations if a more nuanced—and potentially therapeutically beneficial—interpretation of cross-modal activity is to be assessed and applied in clinical populations.

## Supplementary Material

Fullerton_manuscript_revised_Supplementary_Material_bhac277

## References

[ref1a] Aasted CM, Yücel MA, Cooper RJ, Dubb J, Tsuzuki D, Becerra L, Petkov MP, Borsook D, Dan I, Boas DA . Anatomical guidance for functional near-infrared spectroscopy: AtlasViewer tutorial. Neurophoton. 2015:2:020801. 10.1117/1.NPh.2.2.020801 PMC4478785 26157991

[ref1] Adank P . Design choices in imaging speech comprehension: an activation likelihood estimation (ALE) meta-analysis. NeuroImage. 2012:63:1601–1613. 22836181 10.1016/j.neuroimage.2012.07.027

[ref2] Ahissar M, Nahum M, Nelken I, Hochstein S. Reverse hierarchies and sensory learning. Philos Trans R Soc B: Biol Sci. 2009:364(1515):285–299. 10.1098/rstb.2008.0253 PMC2674477 18986968

[ref3] Anderson CA, Lazard DS, Hartley DEH. Plasticity in bilateral superior temporal cortex: effects of deafness and cochlear implantation on auditory and visual speech processing. Hear Res. 2017a:343:138–149. 27473501 10.1016/j.heares.2016.07.013

[ref4] Anderson CA, Wiggins IM, Kitterick PT, Hartley DEH. Adaptive benefit of cross-modal plasticity following cochlear implantation in deaf adults. Proc Natl Acad Sci U S A. 2017b:114:10256–10261. 28808014 10.1073/pnas.1704785114 PMC5617272

[ref5] Barker JW, Aarabi A, Huppert TJ. Autoregressive model based algorithm for correcting motion and serially correlated errors in fNIRS. Biomed Opt Express. 2013:4:1366. 24009999 10.1364/BOE.4.001366 PMC3756568

[ref6] Bates D, Mächler M, Bolker B, Walker S. Fitting linear mixed-effects models using lme4. J Stat Softw. 2015:67:1–48.

[ref7] Bavelier D, Neville HJ. Cross-modal plasticity: where and how? Nat Rev Neurosci. 2002:3:443–452. 12042879 10.1038/nrn848

[ref8] Benjamini Y, Hochberg Y. Controlling the false discovery rate: a practical and powerful approach to multiple testing. J R Stat Soc B (Methodol). 1995:57:289–300.

[ref9] Bernstein LE, Auer ETJ, Jiang J, Eberhardt SP. Auditory perceptual learning for speech perception can be enhanced by audiovisual training. Front Neurosci. 2013:7:34. 10.3389/fnins.2013.00034 PMC3600826 23515520

[ref10] Bernstein LE, Liebenthal E. Neural pathways for visual speech perception. Front Neurosci. 2014:8:386. 10.3389/fnins.2014.00386 PMC4248808 25520611

[ref11] Bizley JK, Nodal FR, Bajo VM, Nelken I, King AJ. Physiological and anatomical evidence for multisensory interactions in auditory cortex. Cereb Cortex. 2007:17(9):2172–2189. 17135481 10.1093/cercor/bhl128 PMC7116518

[ref12] Blamey P, Artieres F, Baskent D, Bergeron F, Beynon A, Burke E, Dillier N, Dowell R, Fraysse B, Gallégo S, et al. Factors affecting auditory performance of postlinguistically deaf adults using cochlear implants: an update with 2251 patients. Audiol Neurotol. 2013:18:36–47. 10.1159/000343189 23095305

[ref13] Boisgontier MP, Cheval B. The anova to mixed model transition. Neurosci Biobehav Rev. 2016:68:1004–1005. 27241200 10.1016/j.neubiorev.2016.05.034

[ref14] Boisvert I, Reis M, Au A, Cowan R, Dowell RC. Cochlear implantation outcomes in adults: a scoping review. PLoS One. 2020:15:e0232421. 32369519 10.1371/journal.pone.0232421 PMC7199932

[ref15] Bolia RS, Nelson WT, Ericson MA, Simpson BD. A speech corpus for multitalker communications research. J Acoust Soc Am. 2000:107:1065–1066. 10687719 10.1121/1.428288

[ref16] Bowyer SM . Coherence a measure of the brain networks: past and present. Neuropsychiatr Electrophysiol. 2016:2:1.

[ref17] Brown VA . An introduction to linear mixed-effects modeling in R. Adv Meth Pract Psychol Sci. 2021:4:2515245920960351.

[ref18] Brungart DS . Informational and energetic masking effects in the perception of two simultaneous talkers. J Acoust Soc Am. 2001:109:1101–1109. 11303924 10.1121/1.1345696

[ref19] Buckley KA, Tobey EA. Cross-modal plasticity and speech perception in pre- and postlingually deaf cochlear implant users. Ear Hear. 2011:32:2–15. 20829699 10.1097/AUD.0b013e3181e8534c

[ref20] Calvert GA, Bullmore ET, Brammer MJ, Campbell R, Williams SCR, McGuire PK, Woodruff PWR, Iversen SD, David AS. Activation of auditory cortex during silent lipreading. Science. 1997:276:593–596. 9110978 10.1126/science.276.5312.593

[ref21] Capek CM, Woll B, MacSweeney M, Waters D, McGuire PK, David AS, Brammer MJ, Campbell R. Superior temporal activation as a function of linguistic knowledge: Insights from deaf native signers who speechread. Brain Lang. 2010:112:129–134. 20042233 10.1016/j.bandl.2009.10.004 PMC3398390

[ref22] Carlyon RP, Goehring T. Cochlear implant research and development in the twenty-first century: a critical update. J Assoc Res Otolaryngol. 2021:22:481–508. 34432222 10.1007/s10162-021-00811-5 PMC8476711

[ref23] Chen L-C, Sandmann P, Thorne JD, Bleichner MG, Debener S. Cross-modal functional reorganization of visual and auditory cortex in adult cochlear implant users identified with fNIRS. Neural Plast. 2016:2016:1–13. 10.1155/2016/4382656 PMC4706950 26819766

[ref24] Chen L-C, Puschmann S, Debener S. Increased cross-modal functional connectivity in cochlear implant users. Sci Rep. 2017:7:10043. 28855675 10.1038/s41598-017-10792-2 PMC5577186

[ref25] Chen L-C, Sandmann P, Thorne JD, Herrmann CS, Debener S. Association of concurrent fNIRS and EEG signatures in response to auditory and visual stimuli. Brain Topogr. 2015:28:710–725. 25589030 10.1007/s10548-015-0424-8

[ref26] Connor CE, Brincat SL, Pasupathy A. Transformation of shape information in the ventral pathway. Curr Opin Neurobiol. 2007:17:140–147. 17369035 10.1016/j.conb.2007.03.002

[ref27] Crosse MJ, Butler JS, Lalor EC. Congruent visual speech enhances cortical entrainment to continuous auditory speech in noise-free conditions. J Neurosci. 2015:35:14195–14204. 26490860 10.1523/JNEUROSCI.1829-15.2015 PMC6605423

[ref28] Curtis CE, Sun FT, Miller LM, D’Esposito M. Coherence between fMRI time-series distinguishes two spatial working memory networks. NeuroImage. 2005:26:177–183. 15862217 10.1016/j.neuroimage.2005.01.040

[ref30] de Kerangal M, Vickers D, Chait M. The effect of healthy aging on change detection and sensitivity to predictable structure in crowded acoustic scenes. Hear Res. 2021:399:108074. 33041093 10.1016/j.heares.2020.108074

[ref31] Dewey RS, Hartley DEH. Cortical cross-modal plasticity following deafness measured using functional near-infrared spectroscopy. Hear Res. 2015:325:55–63. 25819496 10.1016/j.heares.2015.03.007

[ref32] Doucet ME, Bergeron F, Lassonde M, Ferron P, Lepore F. Cross-modal reorganization and speech perception in cochlear implant users. Brain. 2006:129:3376–3383. 17003067 10.1093/brain/awl264

[ref33] Doucet M-E, Gosselin F, Lassonde M, Guillemot J-P, Lepore F. Development of visual-evoked potentials to radially modulated concentric patterns. NeuroReport. 2005:16:1753–1756. 16237321 10.1097/01.wnr.0000185011.91197.58

[ref34] Finney EM, Fine I, Dobkins KR. Visual stimuli activate auditory cortex in the deaf. Nat Neurosci. 2001:4:1171–1173. 11704763 10.1038/nn763

[ref35] Firszt JB, Holden LK, Skinner MW, Tobey EA, Peterson A, Gaggl W, Runge-Samuelson CL, Wackym PA. Recognition of speech presented at soft to loud levels by adult cochlear implant recipients of three cochlear implant systems. Ear Hear. 2004:25:375–387. 15292777 10.1097/01.aud.0000134552.22205.ee

[ref36] Fishburn FA, Ludlum RS, Vaidya CJ, Medvedev AV. Temporal derivative distribution repair (TDDR): a motion correction method for fNIRS. NeuroImage. 2019:184:171–179. 30217544 10.1016/j.neuroimage.2018.09.025 PMC6230489

[ref37] Gifford RH, Shallop JK, Peterson AM. Speech recognition materials and ceiling effects: considerations for cochlear implant programs. Audiol Neurotol. 2008:13:193–205. 10.1159/000113510 18212519

[ref38] Giraud A-L, Lorenzi C, Ashburner J, Wable J, Johnsrude I, Frackowiak R, Kleinschmidt A. Representation of the temporal envelope of sounds in the human brain. J Neurophysiol. 2000:84:1588–1598. 10980029 10.1152/jn.2000.84.3.1588

[ref39] Giraud A-L, Price CJ, Graham JM, Truy E, Frackowiak RSJ. Cross-modal plasticity underpins language recovery after cochlear implantation. Neuron. 2001:30:657–664. 11430800 10.1016/s0896-6273(01)00318-x

[ref40] Gramfort A, Luessi M, Larson E, Engemann DA, Strohmeier D, Brodbeck C, Goj R, Jas M, Brooks T, Parkkonen L, et al. MEG and EEG data analysis with MNE-Python. Front Neurosci. 2013:7:267. 10.3389/fnins.2013.00267 PMC3872725 24431986

[ref41] Gramfort A, Luessi M, Larson E, Engemann DA, Strohmeier D, Brodbeck C, Parkkonen L, Hämäläinen MS. MNE software for processing MEG and EEG data. NeuroImage. 2014:86:446–460. 24161808 10.1016/j.neuroimage.2013.10.027 PMC3930851

[ref42] Gransier R, Luke R, van Wieringen A, Wouters J. Neural modulation transmission is a marker for speech perception in noise in cochlear implant users. Ear Hear. 2020:41:591–602. 31567565 10.1097/AUD.0000000000000783

[ref43] Green KMJ, Bhatt YM, Mawman DJ, O’Driscoll MP, Saeed SR, Ramsden RT, Green MW. Predictors of audiological outcome following cochlear implantation in adults. Cochlear Implants Int. 2007:8:1–11. 17479968 10.1179/cim.2007.8.1.1

[ref44] Hall DA, Fussell C, Summerfield AQ. Reading fluent speech from talking faces: typical brain networks and individual differences. J Cogn Neurosci. 2005:17:939–953. 15969911 10.1162/0898929054021175

[ref45] Heydebrand G, Hale S, Potts L, Gotter B, Skinner M. Cognitive predictors of improvements in adults’ spoken word recognition six months after cochlear implant activation. Audiol Neurotol. 2007:12:254–264. 10.1159/000101473 17406104

[ref46] Hickok G, Poeppel D. The cortical organization of speech processing. Nat Rev Neurosci. 2007:8:393–402. 17431404 10.1038/nrn2113

[ref46a] Hickok G, Poeppel D . Neural basis of speech perception. Handb Clin Neurol. 2015:129:149–160. 25726267 10.1016/B978-0-444-62630-1.00008-1

[ref47] Holden LK, Finley CC, Firszt JB, Holden TA, Brenner C, Potts LG, Gotter BD, Vanderhoof SS, Mispagel K, Heydebrand G, et al. Factors affecting open-set word recognition in adults with cochlear implants. Ear Hear. 2013:34:342–360. 23348845 10.1097/AUD.0b013e3182741aa7 PMC3636188

[ref48] Jasper HH . The ten-twenty electrode system of the international federation. Electroencephalogr Clin Neurophysiol. 1958:10:370–375. 10590970

[ref49] Karas PJ, Magnotti JF, Metzger BA, Zhu LL, Smith KB, Yoshor D, Beauchamp MS. The visual speech head start improves perception and reduces superior temporal cortex responses to auditory speech. elife. 2019:8:e48116. 31393261 10.7554/eLife.48116 PMC6687434

[ref50] Kayser C, Logothetis NK, Panzeri S. Visual enhancement of the information representation in auditory cortex. Curr Biol. 2010:20(1):19–24. 20036538 10.1016/j.cub.2009.10.068

[ref51] Kim M-B, Shim H-Y, Jin SH, Kang S, Woo J, Han JC, Lee JY, Kim M, Cho Y-S, Moon IJ, et al. Cross-modal and intra-modal characteristics of visual function and speech perception performance in postlingually deafened, cochlear implant users. PLoS One. 2016:11:e0148466. 26848755 10.1371/journal.pone.0148466 PMC4743927

[ref52] Konen CS, Kastner S. Two hierarchically organized neural systems for object information in human visual cortex. Nat Neurosci. 2008:11:224–231. 18193041 10.1038/nn2036

[ref53] Kral A . Unimodal and cross-modal plasticity in the ‘deaf’ auditory cortex. Int J Audiol. 2007:46:479–493. 17828664 10.1080/14992020701383027

[ref54] Kral A, Eggermont JJ. What’s to lose and what’s to learn: development under auditory deprivation, cochlear implants and limits of cortical plasticity. Brain Res Rev. 2007:56(1):259–269. 17950463 10.1016/j.brainresrev.2007.07.021

[ref55] Kuznetsova A, Brockhoff PB, Christensen RHB. lmerTest Package: tests in linear mixed effects models. J Stat Softw. 2017:82(1):1–26.

[ref56] Lazard DS, Giraud A-L. Faster phonological processing and right occipito-temporal coupling in deaf adults signal poor cochlear implant outcome. Nat Commun. 2017:8:14872. 28348400 10.1038/ncomms14872 PMC5379061

[ref57] Lee H-J, Truy E, Mamou G, Sappey-Marinier D, Giraud A-L. Visual speech circuits in profound acquired deafness: a possible role for latent multimodal connectivity. Brain. 2007:130:2929–2941. 17906328 10.1093/brain/awm230

[ref58] Lenth R. Emmeans: estimated marginal means, aka least-squares means. R package version 1.5.0. 2020. http://CRAN.R-project.org/package=emmeans.

[ref59] Lomber SG, Meredith MA, Kral A. Cross-modal plasticity in specific auditory cortices underlies visual compensations in the deaf. Nat Neurosci. 2010:13:1421–1427. 20935644 10.1038/nn.2653

[ref60] Luke R, Larson ED, Shader MJ, Innes-Brown H, Yper LV, Lee AKC, Sowman PF, McAlpine D. Analysis methods for measuring passive auditory fNIRS responses generated by a block-design paradigm. Neurophotonics. 2021a:8:025008. 34036117 10.1117/1.NPh.8.2.025008 PMC8140612

[ref61] Luke R, Shader MJ, McAlpine D. Characterization of Mayer wave oscillations in functional near-infrared spectroscopy using a physiologically informed model of the neural power spectra. Neurophotonics. 2021b:8(4):04001-1-9. 10.1117/1.NPh.8.4.041001 PMC8652350 34901310

[ref62] Luke R, Van Deun L, Hofmann M, van Wieringen A, Wouters J. Assessing temporal modulation sensitivity using electrically evoked auditory steady state responses. Hear Res. 2015:324:37–45. 25746913 10.1016/j.heares.2015.02.006

[ref63] Macherey O, Carlyon RP. Cochlear implants. Curr Biol. 2014:24:R878–R884. 25247367 10.1016/j.cub.2014.06.053

[ref63a] MacSweeney M, Amaro E, Calvert GA, Campbell R, David AS, McGuire P, Williams SCR, Woll B, Brammer MJ . Silent speechreading in the absence of scanner noise: an event-related fMRI study. Neuroreport. 2000:11:1729–1733. 10852233 10.1097/00001756-200006050-00026

[ref64] MacSweeney M, Calvert GA, Campbell R, McGuire PK, David AS, Williams SCR, Woll B, Brammer MJ. Speechreading circuits in people born deaf. Neuropsychologia. 2002:40:801–807. 11900730 10.1016/s0028-3932(01)00180-4

[ref65] Mishkin M, Ungerleider LG, Macko KA. Object vision and spatial vision: two cortical pathways. Trends Neurosci. 1983:6:414–417.

[ref66] Müller K, Lohmann G, Bosch V, von Cramon DY. On multivariate spectral analysis of fmri time series. NeuroImage. 2001:14:347–356. 11467908 10.1006/nimg.2001.0804

[ref67] Müller K, Mildner T, Lohmann G, von Cramon DY. Investigating the stimulus-dependent temporal dynamics of the BOLD signal using spectral methods. J Magn Reson Imaging. 2003:17:375–382. 12594729 10.1002/jmri.10268

[ref68] Okada K, Venezia JH, Matchin W, Saberi K, Hickok G, Alain C. An fMRI study of audiovisual speech perception reveals multisensory interactions in auditory cortex. PLoS One. 2013:8:e68959. 23805332 10.1371/journal.pone.0068959 PMC3689691

[ref69] Okada K, Hickok G. Two cortical mechanisms support the integration of visual and auditory speech: a hypothesis and preliminary data. Neurosci Lett. 2009:452:219–223. 19348727 10.1016/j.neulet.2009.01.060 PMC2667381

[ref70] Oldfield RC . The assessment and analysis of handedness: The Edinburgh Inventory. Neuropsychologia. 1971:9:97–113. 5146491 10.1016/0028-3932(71)90067-4

[ref71] Park J-W, Lee G, Kim B-M, Chang Y, Jung Y-J. Analytic signal-based causal network estimator for hemodynamic signal analysis in the brain. J Korean Phys Soc. 2019:74:847–854.

[ref72] Peelle JE . The hemispheric lateralization of speech processing depends on what “speech” is: a hierarchical perspective. Front Hum Neurosci. 2012:6:309. 10.3389/fnhum.2012.00309 PMC3499798 23162455

[ref73] Peelle JE . Listening effort: How the cognitive consequences of acoustic challenge are reflected in brain and behavior. Ear Hear. 2018:39:204–214. 28938250 10.1097/AUD.0000000000000494 PMC5821557

[ref74] Peelle JE, Sommers MS. Prediction and constraint in audiovisual speech perception. Cortex. 2015:68:169–181. 25890390 10.1016/j.cortex.2015.03.006 PMC4475441

[ref75] Peelle JE, Spehar B, Jones MS, McConkey S, Myerson J, Hale S, Sommers MS, Tye-Murray N. Increased connectivity among sensory and motor regions during visual and audiovisual speech perception. J Neurosci. 2022:42:435–442. 34815317 10.1523/JNEUROSCI.0114-21.2021 PMC8802926

[ref76] Pinti P, Tachtsidis I, Hamilton A, Hirsch J, Aichelburg C, Gilbert S, Burgess PW. The present and future use of functional near-infrared spectroscopy (fNIRS) for cognitive neuroscience. Ann N Y Acad Sci. 2020:1464:5–29. 30085354 10.1111/nyas.13948 PMC6367070

[ref77] Quaresima V, Bisconti S, Ferrari M. A brief review on the use of functional near-infrared spectroscopy (fNIRS) for language imaging studies in human newborns and adults. Brain Lang. 2012:121:79–89. 21507474 10.1016/j.bandl.2011.03.009

[ref79] R Core Team . R: A language and environment for statistical computing. Vienna(Austria): R Foundation for Statistical Computing. 2021. http://www.R-project.org/.

[ref80] Rothauser EH, Chapman ND, Guttman N, Nordby KS, Silbiger HR, Urbanek GE, Weinstock M. IEEE recommended practice for speech quality measurements. IEEE Trans Audio Electroacoust. 1969:17:225–246.

[ref81] Rouger J, Lagleyre S, Démonet J-F, Fraysse B, Deguine O, Barone P. Evolution of crossmodal reorganization of the voice area in cochlear-implanted deaf patients. Hum Brain Mapp. 2012:33:1929–1940. 21557388 10.1002/hbm.21331 PMC6870380

[ref82] Saliba J, Bortfeld H, Levitin DJ, Oghalai JS. Functional near-infrared spectroscopy for neuroimaging in cochlear implant recipients. Hear Res. 2016:338:64–75. 26883143 10.1016/j.heares.2016.02.005 PMC4967399

[ref83] Sandmann P, Dillier N, Eichele T, Meyer M, Kegel A, Pascual-Marqui RD, Marcar VL, Jäncke L, Debener S. Visual activation of auditory cortex reflects maladaptive plasticity in cochlear implant users. Brain. 2012:135:555–568. 22232592 10.1093/brain/awr329

[ref84] Santosa H, Zhai X, Fishburn F, Huppert T. The NIRS Brain AnalyzIR Toolbox. Algorithms. 2018:11:73. 10.3390/a11050073 PMC11218834 38957522

[ref85] Sarter M, Givens B, Bruno JP. The cognitive neuroscience of sustained attention: where top-down meets bottom-up. Brain Res Rev. 2001:35:146–160. 11336780 10.1016/s0165-0173(01)00044-3

[ref86] Shader MJ, Luke R, Gouailhardou N, McKay CM. The use of broad vs restricted regions of interest in functional near-infrared spectroscopy for measuring cortical activation to auditory-only and visual-only speech. Hear Res. 2021:406:108256. 34051607 10.1016/j.heares.2021.108256

[ref87] Sherafati A, Dwyer N, Bajracharya A, Hassanpour MS, Eggebrecht AT, Firszt JB, Culver JP, Peelle JE. Prefrontal cortex supports speech perception in listeners with cochlear implants. elife. 2022:11:e75323. 10.7554/eLife.75323 PMC9225001 35666138

[ref88] Skipper JI, Nusbaum HC, Small SL. Listening to talking faces: motor cortical activation during speech perception. NeuroImage. 2005:25:76–89. 15734345 10.1016/j.neuroimage.2004.11.006

[ref89] Sommers MS, Tye-Murray N, Spehar B. Auditory-visual speech perception and auditory-visual enhancement in normal-hearing younger and older adults. Ear Hear. 2005:26:263–275. 15937408 10.1097/00003446-200506000-00003

[ref90] Strelnikov K, Marx M, Lagleyre S, Fraysse B, Deguine O, Barone P. PET-imaging of brain plasticity after cochlear implantation. Hear Res. 2015:322:180–187. 25448166 10.1016/j.heares.2014.10.001

[ref91] Stropahl M, Chen L-C, Debener S. Cortical reorganization in postlingually deaf cochlear implant users: Intra-modal and cross-modal considerations. Hear Res. 2017:343:128–137. 27473503 10.1016/j.heares.2016.07.005

[ref92] Stropahl M, Debener S. Auditory cross-modal reorganization in cochlear implant users indicates audio-visual integration. NeuroImage Clin. 2017:16:514–523. 28971005 10.1016/j.nicl.2017.09.001 PMC5609862

[ref93] Stropahl M, Plotz K, Schönfeld R, Lenarz T, Sandmann P, Yovel G, De Vos M, Debener S. Cross-modal reorganization in cochlear implant users: auditory cortex contributes to visual face processing. NeuroImage. 2015:121:159–170. 26220741 10.1016/j.neuroimage.2015.07.062

[ref94] Sun FT, Miller LM, D’Esposito M. Measuring interregional functional connectivity using coherence and partial coherence analyses of fMRI data. NeuroImage. 2004:21:647–658. 14980567 10.1016/j.neuroimage.2003.09.056

[ref95] Thielen J, Bosch SE, van Leeuwen TM, van Gerven MAJ, van Lier R. Evidence for confounding eye movements under attempted fixation and active viewing in cognitive neuroscience. Sci Rep. 2019:9:17456. 31767911 10.1038/s41598-019-54018-z PMC6877555

[ref96] Tong Y, Lindsey KP, Frederick B, deB. Partitioning of physiological noise signals in the brain with concurrent near-infrared spectroscopy and fMRI. J Cereb Blood Flow Metab. 2011:31:2352–2362. 21811288 10.1038/jcbfm.2011.100 PMC3253380

[ref97] Vigneau M, Beaucousin V, Hervé PY, Duffau H, Crivello F, Houdé O, Mazoyer B, Tzourio-Mazoyer N. Meta-analyzing left hemisphere language areas: phonology, semantics, and sentence processing. NeuroImage. 2006:30:1414–1432. 16413796 10.1016/j.neuroimage.2005.11.002

[ref98] Wilson BS, Dorman MF. Cochlear implants: current designs and future possibilities. J Rehabil Res Dev. 2008:45:695–730. 18816422 10.1682/jrrd.2007.10.0173

[ref100] Wolf U, Toronov V, Choi JH, Gupta R, Michalos A, Gratton E, Wolf M. Correlation of functional and resting state connectivity of cerebral oxy-, deoxy-, and total hemoglobin concentration changes measured by near-infrared spectrophotometry. J Biomed Opt. 2011:16:087013. 21895340 10.1117/1.3615249 PMC3170400

[ref101] Yücel MA, Selb J, Aasted CM, Lin P-Y, Borsook D, Becerra L, Boas DA. Mayer waves reduce the accuracy of estimated hemodynamic response functions in functional near-infrared spectroscopy. Biomed Opt Express. 2016:7:3078–3088. 27570699 10.1364/BOE.7.003078 PMC4986815

[ref102] Zhou X, Seghouane A-K, Shah A, Innes-Brown H, Cross W, Litovsky R, McKay CM. Cortical speech processing in postlingually deaf adult cochlear implant users, as revealed by functional near-infrared spectroscopy. Trends Hear. 2018:22:233121651878685. 10.1177/2331216518786850 PMC6053859 30022732

[ref103] Zion Golumbic EM, Ding N, Bickel S, Lakatos P, Schevon CA, McKhann GM, Goodman RR, Emerson R, Mehta AD, Simon JZ, et al. Mechanisms underlying selective neuronal tracking of attended speech at a “cocktail party”. Neuron. 2013:77:980–991. 23473326 10.1016/j.neuron.2012.12.037 PMC3891478

