# Supplementary material for: Cross-modal functional connectivity supports speech understanding in cochlear implant users
Source: Cereb Cortex. 2022 Aug 20;33(7):3350–71. doi: 10.1093/cercor/bhac277 (PMC10068270; doi:10.1093/cercor/bhac277)
Supplement: Fullerton_manuscript_revised_Supplementary_Material_bhac277 [file fullerton_manuscript_revised_supplementary_material_bhac277.pdf]

Supplementary Material

Supplementary Table 1. Estimates of HbO and HbR activity, Visual only condition. Channels indicating a significant group x stimulus interaction. Model notation: ß ~ Group*Stim + (1| Subject)

	Channel (Source-Detector)	Chroma	beta	SE	tstat	p (FDR)
(Intercept)	1-1	hbo	-0.79	0.27	-2.93	0.02
Group(CI)	1-1	hbo	0.00	0.27	0.01	0.58
Stim(Non-speech)	1-1	hbo	0.66	0.22	3.01	0.01
Group(CI):Stim(Non-speech)	1-1	hbo	-0.56	0.22	-2.59	0.03*
(Intercept)	1-1	hbr	-0.13	0.18	-0.75	0.35
Group (CI)	1-1	hbr	0.25	0.18	1.41	0.18
Stim(Non-speech)	1-1	hbr	0.06	0.10	0.58	0.40
Group(CI):Stim(Non-speech)	1-1	hbr	-0.17	0.10	-1.70	0.13
(Intercept)	1-6	hbo	-1.52	0.27	-5.58	0.00
Group (CI)	1-6	hbo	0.69	0.27	2.53	0.03
Stim(Non-speech)	1-6	hbo	0.92	0.22	4.09	0.00
Group(CI):Stim(Non-speech)	1-6	hbo	-0.69	0.22	-3.08	0.01*
(Intercept)	1-6	hbr	-0.14	0.18	-0.77	0.34
Group(CI)	1-6	hbr	0.01	0.18	0.07	0.57
Stim(Non-speech)	1-6	hbr	0.06	0.11	0.56	0.40
Group(CI):Stim(Non-speech)	1-6	hbr	-0.20	0.11	-1.71	0.13
(Intercept)	2-1	hbo	-1.20	0.26	-4.67	0.00
Group (CI)	2-1	hbo	0.49	0.26	1.90	0.10
Stim(Non-speech)	2-1	hbo	0.77	0.21	3.69	0.00
Group(CI):Stim(Non-speech)	2-1	hbo	-0.64	0.21	-3.06	0.01*
(Intercept)	2-1	hbr	-0.22	0.17	-1.30	0.20
Group (CI)	2-1	hbr	-0.03	0.17	-0.15	0.55
Stim(Non-speech)	2-1	hbr	-0.12	0.11	-1.15	0.23
Group(CI):Stim(Non-speech)	2-1	hbr	-0.32	0.11	-2.94	0.02*
(Intercept)	2-7	hbo	-0.40	0.31	-1.33	0.19
Group (CI)	2-7	hbo	0.28	0.31	0.91	0.29
Stim(Non-speech)	2-7	hbo	0.64	0.25	2.51	0.04
Group(CI):Stim(Non-speech)	2-7	hbo	-0.69	0.25	-2.72	0.02*
(Intercept)	2-7	hbr	-0.27	0.17	-1.57	0.15
Group (CI)	2-7	hbr	0.07	0.17	0.38	0.46
Stim(Non-speech)	2-7	hbr	0.31	0.11	2.92	0.02
Group(CI):Stim(Non-speech)	2-7	hbr	-0.25	0.11	-2.32	0.05
(Intercept)	3-3	hbo	1.21	0.28	4.24	0.00
Group (CI)	3-3	hbo	0.20	0.28	0.69	0.36
Stim(Non-speech)	3-3	hbo	0.43	0.26	1.67	0.14
Group(CI):Stim(Non-speech)	3-3	hbo	0.00	0.26	0.00	0.58
(Intercept)	3-3	hbr	-0.80	0.17	-4.57	0.00
Group (CI)	3-3	hbr	-0.12	0.17	-0.69	0.37
Stim(Non-speech)	3-3	hbr	0.04	0.12	0.30	0.49
Group(CI):Stim(Non-speech)	3-3	hbr	-0.59	0.12	-4.94	0.00**
(Intercept)	4-3	hbo	0.74	0.29	2.54	0.03
Group (CI)	4-3	hbo	0.37	0.29	1.28	0.20
Stim(Non-speech)	4-3	hbo	0.80	0.25	3.19	0.01
Group(CI):Stim(Non-speech)	4-3	hbo	-0.35	0.25	-1.41	0.18

**(Intercept)**	4-3	hbr	-0.82	0.18	-4.51	0.00
**Group(CI)**	4-3	hbr	0.10	0.18	0.52	0.42
**Stim(Non-speech)**	4-3	hbr	0.17	0.11	1.49	0.16
**Group(CI):Stim(Non-speech)**	4-3	hbr	-0.27	0.11	-2.45	0.04*
**(Intercept)**	4-4	hbo	1.37	0.29	4.66	0.00
**Group (CI)**	4-4	hbo	0.40	0.29	1.35	0.19
**Stim(Non-speech)**	4-4	hbo	0.90	0.25	3.55	0.00
**Group(CI):Stim(Non-speech)**	4-4	hbo	-0.25	0.25	-0.97	0.28
**(Intercept)**	4-4	hbr	-1.14	0.18	-6.27	0.00
**Group (CI)**	4-4	hbr	0.04	0.18	0.21	0.52
**Stim(Non-speech)**	4-4	hbr	0.16	0.12	1.33	0.19
**Group(CI):Stim(Non-speech)**	4-4	hbr	-0.35	0.12	-2.93	0.02*
**(Intercept)**	5-5	hbo	-0.82	0.30	-2.68	0.03
**Group (CI)**	5-5	hbo	-0.15	0.30	-0.49	0.43
**Stim(Non-speech)**	5-5	hbo	0.43	0.27	1.59	0.15
**Group(CI):Stim(Non-speech)**	5-5	hbo	-0.65	0.27	-2.42	0.04*
**(Intercept)**	5-5	hbr	-0.07	0.19	-0.35	0.48
**Group (CI)**	5-5	hbr	0.18	0.19	0.93	0.29
**Stim(Non-speech)**	5-5	hbr	0.15	0.13	1.11	0.24
**Group(CI):Stim(Non-speech)**	5-5	hbr	-0.06	0.13	-0.44	0.45
**(Intercept)**	6-11	hbo	-0.88	0.27	-3.23	0.01
**Group (CI)**	6-11	hbo	0.55	0.27	2.01	0.08
**Stim(Non-speech)**	6-11	hbo	0.85	0.23	3.69	0.00
**Group(CI):Stim(Non-speech)**	6-11	hbo	-0.77	0.23	-3.31	0.01*
**(Intercept)**	6-11	hbr	-0.06	0.19	-0.34	0.48
**Group (CI)**	6-11	hbr	-0.08	0.19	-0.41	0.46
**Stim(Non-speech)**	6-11	hbr	-0.16	0.13	-1.29	0.20
**Group(CI):Stim(Non-speech)**	6-11	hbr	-0.12	0.13	-0.97	0.28
**(Intercept)**	8-2	hbo	0.61	0.33	1.86	0.11
**Group (CI)**	8-2	hbo	0.50	0.33	1.52	0.16
**Stim(Non-speech)**	8-2	hbo	0.78	0.29	2.71	0.02
**Group(CI):Stim(Non-speech)**	8-2	hbo	-0.80	0.29	-2.78	0.02*
**(Intercept)**	8-2	hbr	-0.91	0.18	-5.14	0.00
**Group (CI)**	8-2	hbr	-0.25	0.18	-1.41	0.18
**Stim(Non-speech)**	8-2	hbr	-0.10	0.12	-0.81	0.33
**Group(CI):Stim(Non-speech)**	8-2	hbr	-0.17	0.12	-1.41	0.18
**(Intercept)**	8-8	hbo	2.84	0.36	7.82	0.00
**Group (CI)**	8-8	hbo	0.26	0.36	0.72	0.35
**Stim(Non-speech)**	8-8	hbo	-0.44	0.32	-1.40	0.18
**Group(CI):Stim(Non-speech)**	8-8	hbo	-1.57	0.32	-4.94	0.00**
**(Intercept)**	8-8	hbr	-1.14	0.19	-5.88	0.00
**Group (CI)**	8-8	hbr	0.13	0.19	0.65	0.37
**Stim(Non-speech)**	8-8	hbr	0.51	0.14	3.56	0.00
**Group(CI):Stim(Non-speech)**	8-8	hbr	-0.27	0.14	-1.90	0.10
**(Intercept)**	9-8	hbo	3.12	0.31	10.22	0.00
**Group (CI)**	9-8	hbo	0.33	0.31	1.10	0.25
**Stim(Non-speech)**	9-8	hbo	0.41	0.26	1.56	0.16
**Group(CI):Stim(Non-speech)**	9-8	hbo	-1.51	0.26	-5.70	0.00**
**(Intercept)**	9-8	hbr	-1.17	0.18	-6.60	0.00
**Group (CI)**	9-8	hbr	-0.23	0.18	-1.31	0.20
**Stim(Non-speech)**	9-8	hbr	0.05	0.13	0.41	0.46
**Group(CI):Stim(Non-speech)**	9-8	hbr	-0.12	0.13	-0.92	0.29
**(Intercept)**	9-9	hbo	1.15	0.30	3.83	0.00

**Group (CI)**	9-9	hbo	0.34	0.30	1.14	0.24
**Stim(Non-speech)**	9-9	hbo	0.58	0.26	2.23	0.06
**Group(CI):Stim(Non-speech)**	9-9	hbo	-0.91	0.26	-3.46	0.00**
**(Intercept)**	9-9	hbr	-0.78	0.18	-4.43	0.00
**Group (CI)**	9-9	hbr	-0.14	0.18	-0.77	0.34
**Stim(Non-speech)**	9-9	hbr	0.12	0.11	1.10	0.24
**Group(CI):Stim(Non-speech)**	9-9	hbr	0.09	0.11	0.81	0.33
**(Intercept)**	10-4	hbo	0.58	0.34	1.71	0.13
**Group (CI)**	10-4	hbo	0.76	0.34	2.22	0.06
**Stim(Non-speech)**	10-4	hbo	0.66	0.30	2.15	0.07
**Group(CI):Stim(Non-speech)**	10-4	hbo	-0.93	0.30	-3.06	0.01*
**(Intercept)**	10-4	hbr	-0.72	0.18	-4.02	0.00
**Group (CI)**	10-4	hbr	-0.13	0.18	-0.74	0.35
**Stim(Non-speech)**	10-4	hbr	0.05	0.12	0.41	0.46
**Group(CI):Stim(Non-speech)**	10-4	hbr	-0.36	0.12	-3.08	0.01*
**(Intercept)**	10-9	hbo	2.99	0.44	6.86	0.00
**Group (CI)**	10-9	hbo	-0.16	0.44	-0.37	0.47
**Stim(Non-speech)**	10-9	hbo	-0.63	0.41	-1.55	0.16
**Group(CI):Stim(Non-speech)**	10-9	hbo	-1.22	0.41	-3.00	0.01*
**(Intercept)**	10-9	hbr	-1.41	0.21	-6.83	0.00
**Group (CI)**	10-9	hbr	0.21	0.21	1.02	0.27
**Stim(Non-speech)**	10-9	hbr	0.41	0.16	2.54	0.03
**Group(CI):Stim(Non-speech)**	10-9	hbr	-0.18	0.16	-1.11	0.24
**(Intercept)**	11-5	hbo	-0.92	0.31	-2.99	0.01
**Group (CI)**	11-5	hbo	0.03	0.31	0.08	0.57
**Stim(Non-speech)**	11-5	hbo	0.33	0.26	1.26	0.21
**Group(CI):Stim(Non-speech)**	11-5	hbo	-0.96	0.26	-3.71	0.00**
**(Intercept)**	11-5	hbr	-0.44	0.19	-2.34	0.05
**Group(CI)**	11-5	hbr	0.35	0.19	1.89	0.10
**Stim(Non-speech)**	11-5	hbr	0.28	0.12	2.26	0.06
**Group(CI):Stim(Non-speech)**	11-5	hbr	-0.09	0.12	-0.74	0.35
**(Intercept)**	11-10	hbo	-0.28	0.33	-0.85	0.31
**Group (CI)**	11-10	hbo	0.13	0.33	0.40	0.46
**Stim(Non-speech)**	11-10	hbo	-0.24	0.30	-0.81	0.33
**Group(CI):Stim(Non-speech)**	11-10	hbo	-0.81	0.30	-2.76	0.02*
**(Intercept)**	11-10	hbr	-0.51	0.20	-2.62	0.03
**Group (CI)**	11-10	hbr	0.19	0.20	0.95	0.29
**Stim(Non-speech)**	11-10	hbr	0.26	0.14	1.83	0.11
**Group(CI):Stim(Non-speech)**	11-10	hbr	-0.16	0.14	-1.11	0.24
**(Intercept)**	11-11	hbo	0.14	0.26	0.52	0.42
**Group (CI)**	11-11	hbo	0.41	0.26	1.57	0.15
**Stim(Non-speech)**	11-11	hbo	-0.17	0.22	-0.77	0.34
**Group(CI):Stim(Non-speech)**	11-11	hbo	-0.85	0.22	-3.90	0.00**
**(Intercept)**	11-11	hbr	-0.37	0.18	-2.07	0.08
**Group (CI)**	11-11	hbr	0.49	0.18	2.75	0.02
**Stim(Non-speech)**	11-11	hbr	0.15	0.13	1.15	0.23
**Group(CI):Stim(Non-speech)**	11-11	hbr	-0.18	0.13	-1.43	0.18
**(Intercept)**	12-6	hbo	0.53	0.35	1.54	0.16
**Group (CI)**	12-6	hbo	0.38	0.35	1.09	0.25
**Stim(Non-speech)**	12-6	hbo	-1.07	0.29	-3.66	0.00
**Group(CI):Stim(Non-speech)**	12-6	hbo	-0.79	0.29	-2.71	0.02*
**(Intercept)**	12-6	hbr	-0.73	0.24	-3.02	0.01
**Group(CI)**	12-6	hbr	-0.19	0.24	-0.81	0.33

**Stim(Non-speech)**	12-6	hbr	0.34	0.19	1.79	0.12
**Group(CI):Stim(Non-speech)**	12-6	hbr	-0.38	0.19	-2.00	0.08
**(Intercept)**	13-12	hbo	-0.26	0.30	-0.86	0.31
**Group(CI)**	13-12	hbo	0.51	0.30	1.73	0.13
**Stim(Non-speech)**	13-12	hbo	0.32	0.27	1.19	0.22
**Group(CI):Stim(Non-speech)**	13-12	hbo	-0.92	0.27	-3.42	0.01*
**(Intercept)**	13-12	hbr	-0.42	0.18	-2.31	0.05
**Group(CI)**	13-12	hbr	0.05	0.18	0.30	0.49
**Stim(Non-speech)**	13-12	hbr	0.15	0.14	1.14	0.24
**Group(CI):Stim(Non-speech)**	13-12	hbr	-0.07	0.14	-0.51	0.43
**(Intercept)**	15-9	hbo	-0.66	0.40	-1.63	0.14
**Group(CI)**	15-9	hbo	-0.67	0.40	-1.66	0.14
**Stim(Non-speech)**	15-9	hbo	-0.96	0.37	-2.57	0.03
**Group(CI):Stim(Non-speech)**	15-9	hbo	-1.36	0.37	-3.66	0.00**
**(Intercept)**	15-9	hbr	0.02	0.25	0.10	0.57
**Group(CI)**	15-9	hbr	0.35	0.25	1.42	0.18
**Stim(Non-speech)**	15-9	hbr	0.47	0.20	2.28	0.05
**Group(CI):Stim(Non-speech)**	15-9	hbr	0.37	0.20	1.82	0.11
**(Intercept)**	15-15	hbo	-0.56	0.40	-1.39	0.18
**Group(CI)**	15-15	hbo	-0.05	0.40	-0.11	0.56
**Stim(Non-speech)**	15-15	hbo	0.13	0.38	0.33	0.48
**Group(CI):Stim(Non-speech)**	15-15	hbo	-1.03	0.38	-2.71	0.02*
**(Intercept)**	15-15	hbr	-0.55	0.24	-2.33	0.05
**Group(CI)**	15-15	hbr	0.22	0.24	0.93	0.29
**Stim(Non-speech)**	15-15	hbr	0.21	0.21	1.00	0.27
**Group(CI):Stim(Non-speech)**	15-15	hbr	0.00	0.21	-0.02	0.58
**(Intercept)**	16-15	hbo	-0.71	0.29	-2.40	0.04
**Group(CI)**	16-15	hbo	0.15	0.29	0.50	0.43
**Stim(Non-speech)**	16-15	hbo	0.31	0.26	1.20	0.22
**Group(CI):Stim(Non-speech)**	16-15	hbo	-1.10	0.26	-4.26	0.00**
**(Intercept)**	16-15	hbr	-0.66	0.20	-3.25	0.01
**Group(CI)**	16-15	hbr	0.02	0.20	0.08	0.57
**Stim(Non-speech)**	16-15	hbr	0.41	0.17	2.47	0.04
**Group(CI):Stim(Non-speech)**	16-15	hbr	-0.08	0.17	-0.49	0.43
**(Intercept)**	17-11	hbo	0.51	0.34	1.51	0.16
**Group(CI)**	17-11	hbo	0.60	0.34	1.79	0.12
**Stim(Non-speech)**	17-11	hbo	-0.33	0.30	-1.09	0.25
**Group(CI):Stim(Non-speech)**	17-11	hbo	-0.82	0.30	-2.71	0.02*
**(Intercept)**	17-11	hbr	-0.67	0.22	-3.02	0.01
**Group(CI)**	17-11	hbr	-0.06	0.22	-0.28	0.50
**Stim(Non-speech)**	17-11	hbr	0.46	0.17	2.66	0.03
**Group(CI):Stim(Non-speech)**	17-11	hbr	-0.33	0.17	-1.89	0.10
**(Intercept)**	17-16	hbo	-0.64	0.29	-2.21	0.06
**Group(CI)**	17-16	hbo	0.27	0.29	0.93	0.29
**Stim(Non-speech)**	17-16	hbo	0.40	0.26	1.54	0.16
**Group(CI):Stim(Non-speech)**	17-16	hbo	-0.47	0.26	-1.83	0.11
**(Intercept)**	17-16	hbr	-0.32	0.19	-1.68	0.14
**Group(CI)**	17-16	hbr	0.24	0.19	1.30	0.20
**Stim(Non-speech)**	17-16	hbr	0.23	0.14	1.71	0.13
**Group(CI):Stim(Non-speech)**	17-16	hbr	-0.48	0.14	-3.58	0.00**

Stim: Stimulus (Speech or Non-speech); Group(NH) and Stim(Speech) are on the intercept.
Significance codes: ***p<0.001, **p<0.01,*p<0.05

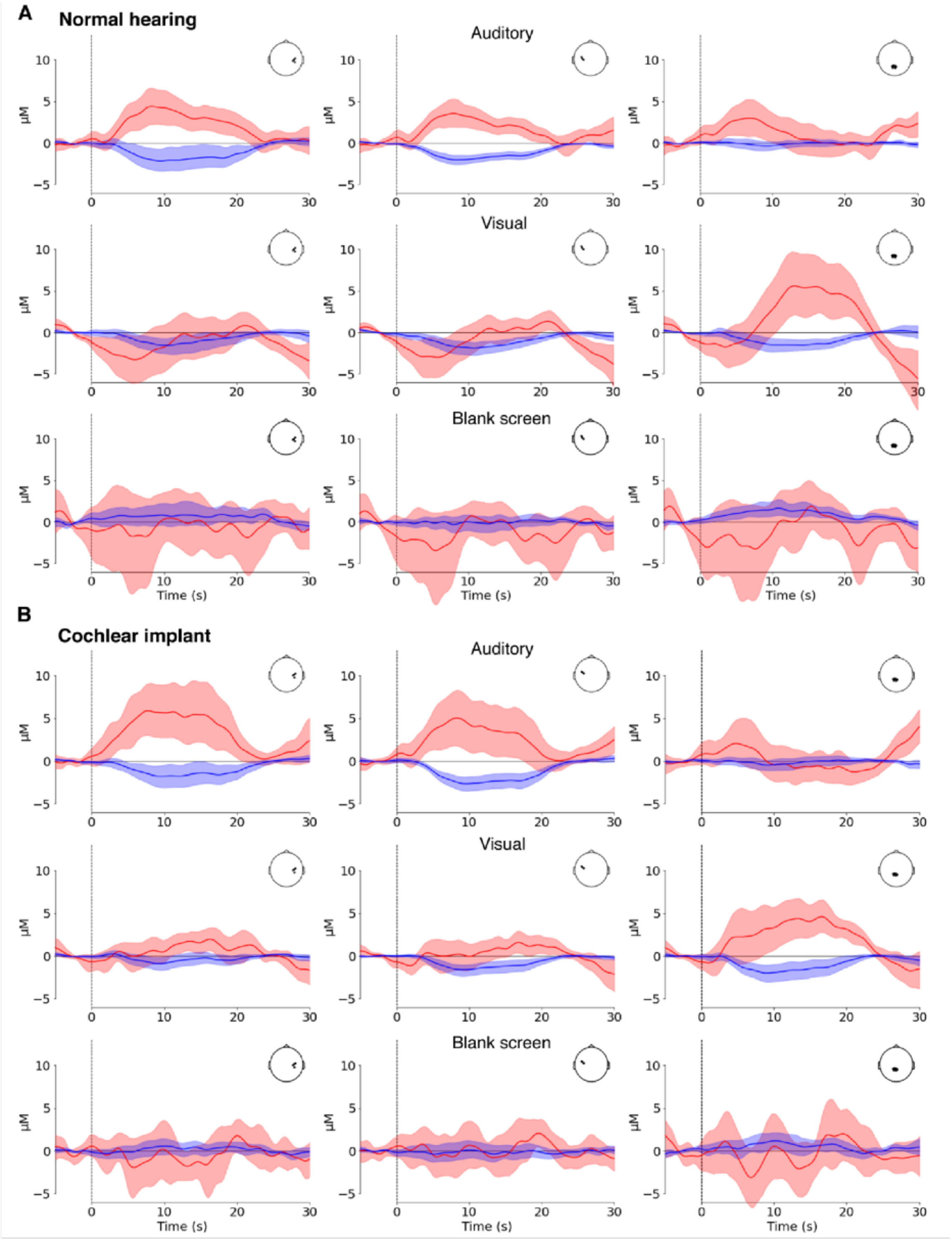

Supplementary Figure 1: Time courses of fNIRS responses to speech stimuli in NH and CI users in the left, right and visual regions of interest (ROI). Epoched, grand-average responses to speech stimuli, processed for initial qualitative inspection, at selected channels in auditory and visual ROIs in response to the auditory, visual, and blank screen conditions in A. NH subjects and B. CI users.

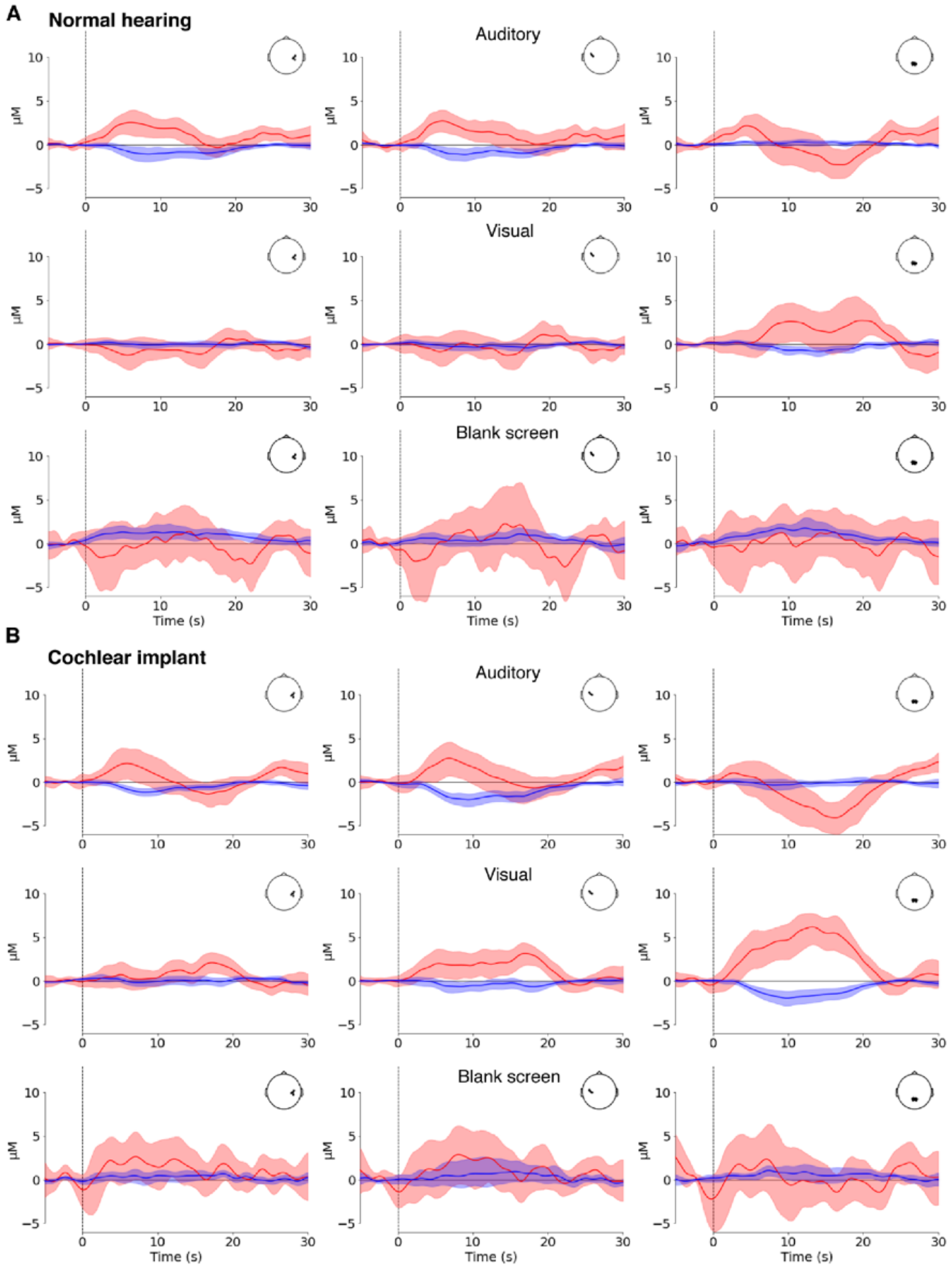

Supplementary Figure 2: Time courses of fNIRS responses to non-speech stimuli in NH and CI users in the left, right and visual regions of interest (ROI). Epoched, grand-average responses to non-speech stimuli, processed for initial qualitative inspection, at selected channels in auditory and visual ROIs in response to the auditory, visual, and blank screen conditions in A. NH subjects and B. CI users.

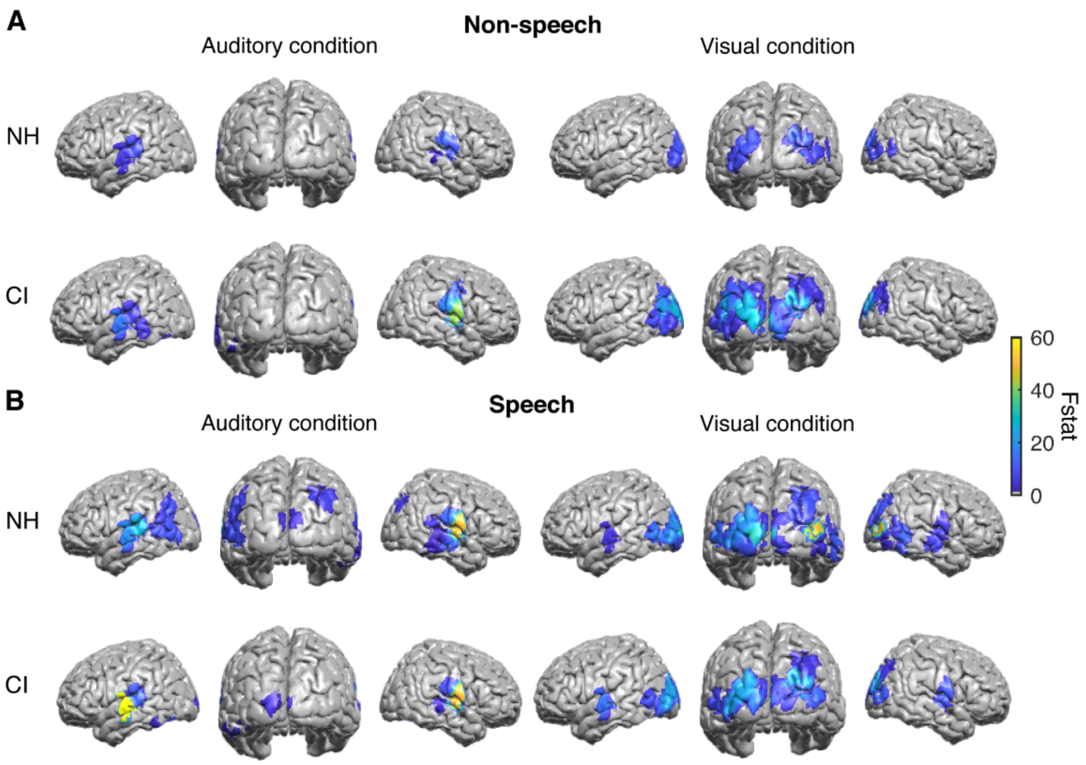

Supplementary Figure 3. Subset analysis of subjects receiving right-sided auditory stimulation only, matched for age, sex and handedness (n=9 CI, n=9 NH). Projection of F-statistic (Fstat) maps to cortex following group-level analysis, showing significant Hotelling's T^2 test results, quantifying the joint activity of oxy-(HbO) and deoxy-hemoglobin (HbR) for both NH and CI participants (corrected p value <0.05) in response to A. Non-speech stimuli and B. Speech stimuli, in both auditory and visual conditions.
